# Aerospace nurses’ competencies in disaster situations: a scoping review

**DOI:** 10.1590/1518-8345.7421.4326

**Published:** 2024-09-23

**Authors:** Bernardo Arnulpho Coelho de Paula, Débora Fernanda Haberland, Fábio José de Almeida Guilherme, Bruno Leal Barbosa, Alexandre Barbosa de Oliveira, Thiago Augusto Soares Monteiro da Silva

**Affiliations:** ^1^ Universidade de Vassouras, Curso de Enfermagem, Vassouras, RJ, Brazil.; ^2^ Universidade Federal do Rio de Janeiro, Escola de Enfermagem Anna Nery, Rio de Janeiro, RJ, Brazil.; ^3^ Força Aérea Brasileira, Instituto de Medicina Aeroespacial Brigadeiro Médico Roberto Teixeira, Rio de Janeiro, RJ, Brazil.

**Keywords:** Nursing, Air Ambulances, Science of Disaster, Aerospace Medicine, Emergencies, Disaster Team

## Abstract

**Objective:**

to map the competencies of aerospace nurses in disaster situations.

**Method:**

a scoping review following the steps recommended by the JBI and the Preferred Reporting Items for Systematic reviews and Meta-Analyses extension for Scoping Reviews (PRISMA-ScR) checklist. The review was conducted in three phases by two independent reviewers, with blinding, and supported by a third reviewer to resolve disagreements. Selection was based on the analysis of titles, descriptors, and abstracts, with specific eligibility criteria, followed by the full-text reading. At the end of the selection phase, 37 publications were included.

**Results:**

the results highlighted the need for the development of technical skills, knowledge of flight physiology, familiarity with aeronautical emergency procedures, communication skills, leadership, and responsibilities in aircraft preparation. During transport, nurses perform patient history taking (anamnesis), physical examinations, patient monitoring, clinical procedures, and manage in-flight complications. After the flight, they conduct documentation, develop procedures, sanitize clinical equipment, and replenish consumable materials.

**Conclusion:**

given the complexity of aerospace nursing practices in disaster situations, it is essential for professionals to develop competencies to ensure safe and effective care. There is a need to develop technologies, regulatory frameworks, and legal provisions for legal support, as well as future studies to validate and deepen the mapped competencies.

## Highlights

**(1)** Need for technical skills and knowledge of flight physiology.

**(2)** Emphasis on the complexity of aerospace nursing in disaster scenarios.

**(3)** Urgency in assessing responsibilities in preparing aircraft.

**(4)** Familiarity with aeronautical emergency procedures is essential.

## Introduction

 Disasters are phenomena resulting from adverse events, which generally impact a susceptible ecosystem, causing harm to humans, material assets, and the environment ^(^ ^1^ ^)^ . Whether they are of natural, technological, or social origin, these events have their risks amplified by human exposure and the combination of vulnerabilities and compromised preparedness and response capacity ^(^ ^1^ ^)^ . In such situations, it is common practice to demand aircraft that assist in accessing affected areas, speeding up health care, and providing humanitarian aid to impacted communities ^(^ ^2^ ^)^ . 

 The aerial rescue and evacuation service began during the Franco-Prussian War (1870) using non-steerable balloons ^(^ ^3^ ^)^ . Subsequently, the invention of airplanes enabled the evolution of this practice during World War I (1914-1918), albeit in a rudimentary manner, as patients were transported in compartments in front of the pilot without the accompaniment of health care professionals. The purpose was the swift removal to a safe location where some form of assistance was available ^(^ ^3^ ^)^ . 

 In World War II (1939-1945), American Nursing saw significant advancements, as wounded individuals began to be transported in cargo planes with three beds each, receiving care from “flight nurses” (flight Nightingales) ^(^ ^4^ ^-^ ^6^ ^)^ . Later, in Brazil, aeromedical evacuation began to be systematized by the Military Fire Brigade of the State of Rio de Janeiro, whose rescue and salvage activities started in 1988 ^(^ ^3^ ^-^ ^4^ ^)^ . 

 In practice, this type of patient transport involves the use of different aircraft, either fixed-wing (airplanes) or rotary-wing (helicopters), the latter commonly employed for faster transport over short distances or to areas that are difficult to access ^(^ ^7^ ^)^ . 

 In more complex contexts, such as public health emergencies and disasters—whether natural, technological, or social in origin—air transport for patient evacuation aims to save as many lives as possible, as well as provide support to hospitals with a high volume of victims requiring care ^(^ ^8^ ^)^ . 

 Given this, there is a highlighted need for the training/preparation of nurses for this type of activity, as these professionals typically make up a significant part of aerospace transport health teams ^(^ ^9^ ^-^ ^10^ ^)^ . In addition to direct care, the nurse’s management role in disasters is crucial for reducing potential health risks and harms that could affect both the victims and the team itself ^(^ ^11^ ^)^ . 

 Studies on the competencies of nurses in disaster situations have been conducted globally ^(^ ^2^ ^,^ ^8^ ^-^ ^12^ ^)^ . This trend underscores the need for an expanded discussion on the topic and the incorporation of evidence on the development of specific competencies to be applied in various types of disasters. This implies the need for continuous review and improvement of training strategies to ensure greater operational effectiveness of missions and a more assertive, systematic response, guided by care and management actions supported by nursing science ^(^ ^9^ ^,^ ^13^ ^)^ . 

Therefore, the study is justified by the necessity to explore this emerging and constantly evolving field, to ensure greater safety and effectiveness of care, to enhance training programs, to develop practices and policies, and to encourage future research. As a starting point, a preliminary search was conducted in December 2023 in MEDLINE (via PubMed), JBI Evidence Synthesis, PROSPERO, and Cochrane Database of Systematic Reviews. No ongoing or published systematic or scoping reviews were found regarding the competencies of aerospace nurses in disaster situations.

In light of the above, the following objective was outlined: to map the competencies of aerospace nurses in disaster situations.

## Method

### Type of study

 This is a scoping review conducted in accordance with the JBI recommendations ^(^ ^14^ ^)^ , with the research protocol registered on the Open Science Framework: osf.io/rh2t6 e DOI: 10.17605/OSF.IO/BRY5Q. 

 The review question was: what are the competencies of the aerospace nurse in the context of disasters? The PCC mnemonic (P – Population, C – Concept and C – Context) was defined as: Population: Nurses working in the aerospace setting; Concept: Competencies, encompassing the mobilization of knowledge, skills, and attitudes necessary for performing specific activities or functions ^(^ ^12^ ^)^ ; Context: Air transportation in disasters, whether of any typology (natural, technological, or social). 

 Through these elements, controlled vocabularies were used: Health Sciences Descriptors (DeCS), Medical Subject Headings (MeSH) Emtree and (Embase subject headings). After conducting the preliminary search, additional terms identified in the titles, abstracts, and descriptors/MeSH of the articles were added ( Figure 1 ). 

### Eligibility criteria

Technical-scientific information sources addressing the competencies of aerospace nurses in disaster situations were considered, in accordance with the PCC mnemonic and the review question.

Studies available in full text were included without temporal or language limitations, and without the requirement for open access to sources, with the objective of broaden the scope of the research.

Publications of any nature were considered, including those derived from qualitative, quantitative, and mixed-method approaches. Thus, primary studies, experimental and quasi-experimental designs, reviews, before-and-after studies, observational studies, time-series studies, cohort studies, cross-sectional studies, and case-control studies were all considered.

Grey literature studies (theses and dissertations databases, guidelines, protocols, websites, opinions, and guidelines) were also included. Books, book chapters, and editorials, as well as duplicated publications, were excluded.

### Period

Searches were conducted from October to December 2023.

### Information sources

The searches were conducted on the Regional Portal of the Brazilian Virtual Health Library (BVS), under the responsibility of the Latin American and Caribbean Center on Health Sciences Information (BIREME). This portal includes databases such as: Spanish Bibliographic Index of Health Sciences (IBECS), Nursing Database (BDENF), Latin American and Caribbean Literature on Health Sciences (LILACS), Peruvian Network of Health Libraries (LIPECS), Scientific Electronic Library Online (SciELO), Medical Literature Analysis and Retrieval System Online via PubMed from the National Library of Medicine (NLM), among others.

Through the CAPES Periodicals Portal, the following databases were accessed: Ebsco: Cummulative Index to Nursing and Allied Health Literature (CINAHL) and Academic Search Premier (ASP); Elsevier: Embase and Scopus; Clarivate Analytics: Web of Science Additionally, CAB Direct (a platform that allows searching in CAB Abstracts) and Global Health were also accessed.

The search also included websites related to aerospace nursing, aeromedical rescue, professional legislation, the International Council of Nurses (ICN) website, internet search engines, and digital libraries containing theses and dissertations.

 To systematize the search through grey literature, the Science.gov portal, an integrator and grey literature repository, was considered: USA.gov, *Epistemonikos* : Database of the Best Evidence-Based Health Care, information Technologies and a network of experts, National Institute for Health and Care Excellence (NICE). 

### Search strategy

The searches were conducted in three stages by two reviewers independently. The blinding process between them was maintained. The research was supported by a third reviewer to resolve disagreements, as well as a librarian affiliated with a federal public university to provide guidance and oversee the process.

 The analysis of titles, abstracts, and descriptors was carried out in the first stage through an initial search in the Medical Literature Analysis and Retrieval System Online (Medline). The boolean operators “AND” and “OR” were used in conjunction with keywords and descriptors ( Figure 1 ). 

**Figure 1:** - Search strategies applied in Medline

**Research**	**Search strategy**	**Results**
#01	**Search:** ”Nursing”[mh] OR Nursing*[tiab] OR “Nurses”[mh] ORNurse*[tiab] Sort by: Most Recent	690.130
#02	**Search:** ”Air Ambulances”[mh] OR Air Ambulance*[tiab] OR Emergency Helicopter*[tiab] OR Helicopter Ambulance*[tiab] OR “Aircraft”[mh] OR Aircraft*[tiab] OR Helicopter*[tiab] OR Airplane*[tiab] OR “Transportation of Patients”[mh] OR Patients Transportation*[tiab] OR Aeromedical evacuation[tiab] OR Airport*[tiab] OR “Aerospace Medicine”[mh] OR “Aviation Medicine”[tiab] OR “Space Medicine”[tiab] OR aerospace[tiab] OR Air[tiab] OR helicopter*[tiab] OR aircraft*[tiab] OR airplane*[tiab] Sort by: Most Recent	362.543
#03	**Search:** ”Disasters”[mh] OR Disaster*[tiab] OR Emergencies[mh] OR Emergenc*[tiab] OR Biological Disaster*[tiab] OR “Mass Casualty Incidents”[mh] OR Mass Casualty Incident*[tiab] OR Mass Casualt*[tiab] OR terror[tiab] OR Bioterrorism[tiab] OR Terrorism[tiab] Sort by: Most Recent	594.755
#04	**Search:** #01 AND #02 AND #03 Sort by: Most Recent	888

The second stage was developed from the comprehensive search in all previously selected databases and repositories.

The third stage, in turn, involved analyzing the reference lists of all sources that met the eligibility criteria. Furthermore, additional information was sought by contacting the authors of the primary studies, including to gain access to other studies on the topic.

### Source selection

 After conducting the searches, all identified citations were imported into the Rayyan ^®^ application (Qatar Computing Research Institute, Doha, Qatar). Initially, the data were analyzed through (re)readings of titles, descriptors, and abstracts, following the eligibility criteria. Excluded studies were presented in the Preferred Reporting Items for Systematic reviews and Meta-Analyses extension for Scoping Reviews (PRISMA-ScR) ^(^ ^15^ ^)^ flowchart regarding the established population, concept, and context. 

The EndNote Web manager (Clarivate Analytics, PA, USA) was used for organizing references and identifying duplicate studies.

Both researchers read the texts in full, which were archived in digital folders. The results obtained from the selection were presented in a flowchart format, based on the PRISMA-ScR model.

### Data extraction

After reading the full texts, the selected data were extracted using an adapted instrument, following the JBI recommendations.

To operationalize the instrument, a pilot test was conducted on three sources to familiarize the reviewers with the selection process, extraction, and data retrieval. During this process, doubts were identified, and some adjustments were made.

### Data analysis and presentation

 From the extracted data, an inductive content analysis was performed ^(^ ^14^ ^)^ . The information reported included the article/document title, authors (name, qualification, profession, institution, country), descriptors (keywords), type of information source, language, objectives, study design and approach, origin and typology of the disaster situation, and competencies of aerospace nursing in the context of disasters (in the pre-flight, flight, and post-flight phases). 

The synthesis of the results was presented in the form of flowcharts, tables, and imagery diagrams, ensuring proper alignment with the objective and the research question.

## Results

**Figure 2:**
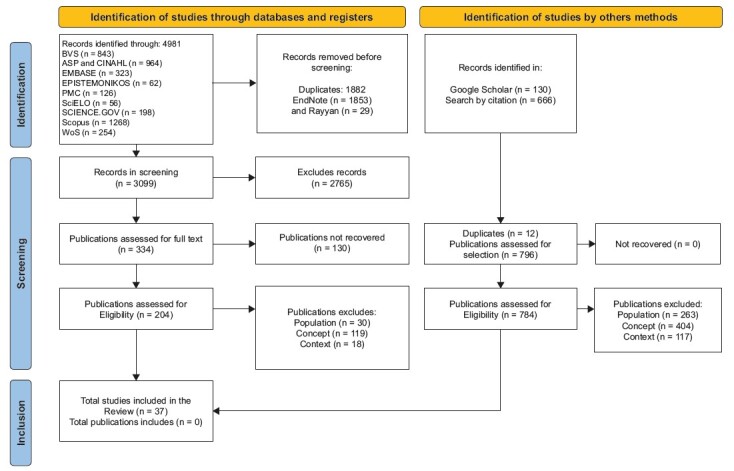
- PRISMA-ScR flowchart

 A total of 4,981 publications were identified, with 1,882 duplicates. Therefore, 3,099 publications were imported into the Rayyan software. After double-blind selection by reviewers, 2,765 sources were excluded. This resulted in 334 publications for full-text analysis. After paired and blinded analysis, 30 publications were excluded due to population, 152 due to concept, and 18 due to context. This resulted in 37 included articles ( Figure 2 ). 

 Other search strategies retrieved 796 publications. Of these, 130 were found on Google Scholar and 666 through reverse search. Out of this total, 12 were duplicates, and 784 were excluded, with 236 due to population, 404 due to context, and 117 due to concept. Thus, no new publications were included through other search methods, as no sources addressing the topic of aerospace nurse competencies in disaster contexts were found, in accordance with the eligibility criteria. In total, 37 publications were included for review ( Figure 2 ). 

 Of the 37 studies on aerospace nurse competencies in disaster contexts, which have been published since 1991, an average of two to three articles per year was identified. It is noteworthy that in 2010, 2011, 2017, and 2020, three publications were identified each year; while in 2003, 2005, 2007, 2008, 2012, 2013, 2021, 2022, and 2023, two publications occurred per year, as shown in Figure 3 . 

Proportionally, this result may be related to progress that have occurred in the field of Nursing regarding the use of health care practices within aviation systems, especially concerning the increase in the frequency and complexity of disasters, the clearer risk perception regarding such events, and the need for discussion of approaches for multiple victims of complex clinical-surgical cases, which require aeromedical transportation and rescue in such situations.

 It is noteworthy that the advent of the specialty of aerospace Nursing contributes to the need for scientific dissemination of the topic and, consequently, the systematization of this practice. Furthermore, the movement of the ICN, initiated more emphatically in 2009, sought to point out strategies for formalizing nurses’ competencies for disaster response ^(^ ^10^ ^,^ ^16^ ^-^ ^17^ ^)^ . 

 Regarding the country of origin, it was identified that 23 studies originated from the United States of America (USA), 11 from Brazil, one from China, one from Finland, and one from Ireland. Regarding language, 26 were written in English and 11 in Portuguese. The investment in aeromedical transportation and rescue in civilian and military settings in these countries is highlighted ( Figure 3 ). 

 It was identified that seven articles were published in the Air Medical Journal, six in the *Revista Brasileira de Enfermagem* , three in the Critical Care Nursing Clinics, two in Military Medicine, and two in the *Texto & Contexto – Enfermagem* journal ( Figure 3 ). 

**Figure 3:** - Characterization of mapped publications

**No.**	**Year**	**Type of disaster**	**Information source**	**Country**	**Language**
F1 ^(^ ^18^ ^)^	1991	Not specified	*Rev Bras Enferm*	Brazil	Portuguese
F2 ^(^ ^19^ ^)^	1995	Not specified	Air Medical Journal	United States	English
F3 ^(^ ^20^ ^)^	1996	Not specified	Air Medical Journal	United States	English
F4 ^(^ ^21^ ^)^	1997	War	*Revista da Escola de Enfermagem da USP*	Brazil	Portuguese
F5 ^(^ ^22^ ^)^	1999	War	*Acta Paul Enferm*	Brazil	Portuguese
F6 ^(^ ^23^ ^)^	2003	Not specified	*Rev Bras Enferm*	Brazil	Portuguese
F7 ^(^ ^24^ ^)^	2003	Not specified	Critical Care Nursing Clinics	United States	English
F8 ^(^ ^25^ ^)^	2005	Not specified	Critical Care Medicine	United States	English
F9 ^(^ ^26^ ^)^	2005	Tsunami	Scandinavian Journal of Surgery	Finland	English
F10 ^(^ ^27^ ^)^	2007	Not specified	AORN Journal	United States	English
F11 ^(^ ^28^ ^)^	2007	Hurricane Katrina.	Disaster Management & Response	United States	English
F12 ^(^ ^29^ ^)^	2008	Tsunami	Air Medical Journal	United States	English
F13 ^(^ ^30^ ^)^	2008	War	Critical Care Nursing Clinics of North America	United States	English
F14 ^(^ ^31^ ^)^	2010	Not specified	Critical Care Nurse	United States	English
F15 ^(^ ^32^ ^)^	2010	Not specified	Journal of Trauma Nursing| JTN	United States	English
F16 ^(^ ^33^ ^)^	2010	War	International Journal of Nursing Practice	United States	English
F17 ^(^ ^34^ ^)^	2011	Not specified	*Rev Bras Enferm*	Brazil	Portuguese
F18 ^(^ ^35^ ^)^	2011	Not specified	*Texto & Contexto-Enfermagem*	Brazil	Portuguese
F19 ^(^ ^36^ ^)^	2011	Not specified	*Rev Bras Enferm*	Brazil	Portuguese
F20 ^(^ ^37^ ^)^	2012	Not specified	*Rev Bras Enferm*	Brazil	Portuguese
F21 ^(^ ^38^ ^)^	2012	War	Air Medical Journal	United States	English
F22 ^(^ ^39^ ^)^	2013	Not specified	Journal of Emergency Nursing	United States	English
F23 ^(^ ^40^ ^)^	2013	War	Journal of Emergency Nursing	United States	English
F24 ^(^ ^41^ ^)^	2015	War	Journal of Emergency Nursing	United States	English
F25 ^(^ ^42^ ^)^	2017	Not specified	International Emergency Nursing	United States	English
F26 ^(^ ^4^ ^)^	2017	War	*Esc Anna Nery – Revista de Enfermagem*	Brazil	Portuguese
F27 ^(^ ^43^ ^)^	2017	Not specified	Military Medicine	United States	English
F28 ^(^ ^44^ ^)^	2018	QBRN ^*^	Air Medical Journal	United States	English
F29 ^(^ ^45^ ^)^	2019	War and natural disaster	Nurs Outlook	United States	English
F30 ^(^ ^46^ ^)^	2020	Not specified	Military Medicine	United States	English
F31 ^(^ ^47^ ^)^	2020	Not specified	Nurse Leader	United States	English
F32 ^(^ ^48^ ^)^	2020	Not specified	Scandinavian Journal of Trauma, Resuscitation and Emergency Medicine country	Ireland	English
F33 ^(^ ^49^ ^)^	2021	Not specified	Air Medical Journal	United States	English
F34 ^(^ ^9^ ^)^	2021	Not specified	Nursing.	Brazil	Portuguese
F35 ^(^ ^50^ ^)^	2022	Not specified	Hong Kong Journal of Emergency Medicine	China	English
F36 ^(^ ^51^ ^)^	2023	Not specified	Air Medical Journal	United States	English
F37 ^(^ ^7^ ^)^	2023	QBRN ^*^	*Texto & Contexto-Enfermagem*	Brazil	Portuguese

^*^ QBRN [CBRN] = Chemical, biological, radiological, and nuclear

 In terms of the disasters typology, it was identified that 22 articles addressed the context of disasters in general, without specifying it; nine referred to wars, two to events involving chemical, biological, radiological, and nuclear agents (CBRN), two to geological events (tsunamis), one to a meteorological event (hurricane), and one was related to natural origin (without defining the exact sub-typology) ( Figure 3 ). 

This plurality of event types suggests the need for further exploration of the topic at hand, understanding that the specificities of each event may imply practices tailored to particular clinical situations.

Furthermore, understanding the challenges faced and the lessons learned in such events is strategic in assuming disaster risk management actions, which involves thinking about processes, that is, actions to be developed before, during, and after such events. Furthermore, there is also a certain emphasis on war situations and CBRN events among the mapped studies, which calls for reflections on contemporary concerns regarding this type of event.

 In summary, the competencies of aerospace nurses in the context of disasters, which were mapped by this scoping review, were categorized into three categories: pre-flight, flight, and post-flight ( Figure 4 ). 

**Figure 4:** - Aerospace nurse competencies in disaster context

**Phase of Transportation**	**Mapping of Aerospace Nurse Competencies in Disaster Context** 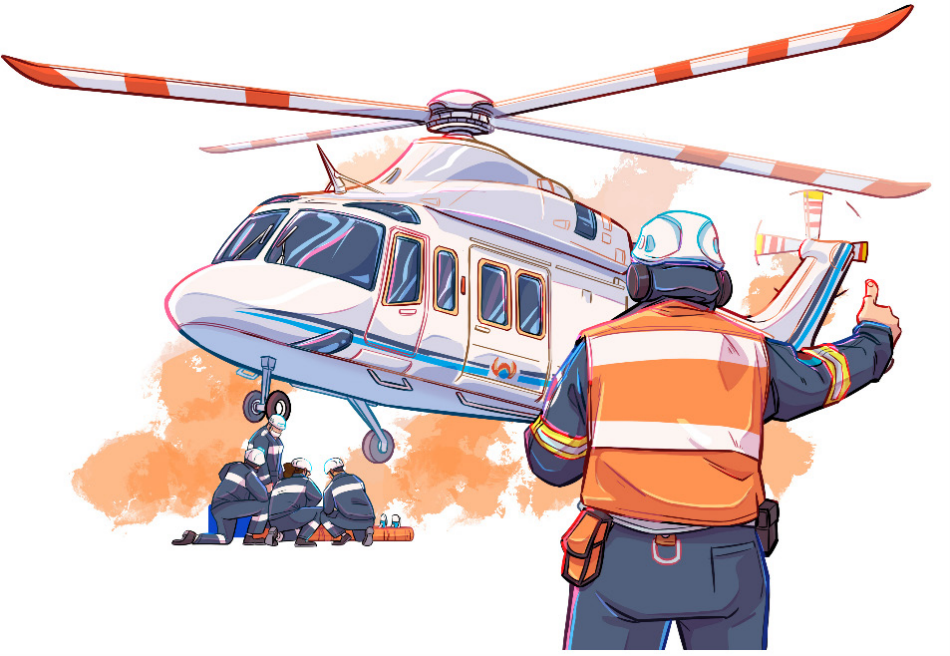
**PRE-FLIGHT**	To know the physiology of flight and aeronautical emergency procedures to provide quality nursing care ^(^ ^7^ ^,^ ^9^ ^,^ ^19^ ^,^ ^33^ ^,^ ^36^ ^-^ ^37^ ^)^ .
	To know flight, patient, and professional safety practices related to aircraft on the ground or in flight ^(^ ^9^ ^,^ ^33^ ^,^ ^36^ ^-^ ^37^ ^,^ ^45^ ^-^ ^48^ ^)^ .
	To know the aircraft used in air transportation and understand the use of related medical equipment ^(^ ^9^ ^,^ ^19^ ^,^ ^24^ ^,^ ^27^ ^,^ ^29^ ^,^ ^33^ ^,^ ^50^ ^)^ .
	To be able to prepare the aircraft for the mission ^(^ ^9^ ^,^ ^19^ ^,^ ^24^ ^,^ ^27^ ^,^ ^46^ ^,^ ^49^ ^-^ ^50^ ^)^ .
	To perform the checklist of standardized materials and medications for use in the aircraft ^(^ ^9^ ^,^ ^19^ ^,^ ^24^ ^,^ ^27^ ^,^ ^49^ ^-^ ^50^ ^)^ .
	To provide the necessary materials, equipment, and oxygen ^(^ ^9^ ^,^ ^19^ ^,^ ^24^ ^,^ ^27^ ^,^ ^49^ ^-^ ^50^ ^)^ .
	To have leadership skills and decision-making ability ^(^ ^28^ ^,^ ^45^ ^)^ .
	To act as a training professional developing programs within the team, to ensure that the team has updated skills, competencies, and knowledge ^(^ ^45^ ^)^ .
	To possess technical skills to deal with disasters, communication skills, and the ability to handle multiple victims ^(^ ^9^ ^,^ ^19^ ^,^ ^24^ ^,^ ^27^ ^,^ ^34^ ^)^ .
	To have good oral and written communication skills, to discuss cases with the team and to complete required reports ^(^ ^7^ ^,^ ^35^ ^,^ ^37^ ^,^ ^45^ ^)^ .
	To assess the need for aircraft use, identifying the nature of the emergency based on the condition, number of victims, and location of the occurrence ^(^ ^9^ ^,^ ^18^ ^,^ ^36^ ^,^ ^44^ ^)^ .
	To plan the entire mission together with the multiprofessional team ^(^ ^22^ ^,^ ^36^ ^,^ ^44^ ^,^ ^51^ ^)^ .
	To conduct patient assessment (Nursing History) and participate in the decision regarding whether the patient should be airlifted or not ^(^ ^20^ ^,^ ^25^ ^,^ ^35^ ^,^ ^38^ ^)^ .
	To perform procedures of various complexities in preparing the patient for transport according to the clinical condition ^(^ ^7^ ^,^ ^9^ ^,^ ^22^ ^-^ ^23^ ^,^ ^25^ ^,^ ^27^ ^-^ ^28^ ^,^ ^30^ ^,^ ^49^ ^-^ ^50^ ^)^ .
**FLIGHT**	To have knowledge about emergency procedures of the aircraft in which the transport is conducted ^(^ ^25^ ^-^ ^26^ ^)^ .
	To have the ability to develop and manage actions of the disaster response plan in an airborne environment ^(^ ^9^ ^,^ ^27^ ^,^ ^39^ ^)^ .
	To possess good written communication skills to make nursing records and notes ^(^ ^21^ ^-^ ^22^ ^)^ .
	To have the capacity to lead the care team during the flight ^(^ ^22^ ^)^ .
	To manage the patient flow, human resources, materials, equipment, and supplies during the flight ^(^ ^9^ ^,^ ^19^ ^,^ ^22^ ^,^ ^26^ ^-^ ^28^ ^,^ ^31^ ^-^ ^32^ ^,^ ^42^ ^,^ ^44^ ^)^ .
	To have the ability to manage incidents during the flight ^(^ ^22^ ^,^ ^36^ ^)^ .
	To use instruments that ensure professional safety and health (PPE and PPE) ^(^ ^7^ ^,^ ^40^ ^)^ .
	To conduct a focused medical history and physical examination, establishing the nursing process ^(^ ^20^ ^,^ ^25^ ^,^ ^36^ ^,^ ^38^ ^-^ ^39^ ^,^ ^41^ ^)^ .
	To provide psychosocial support to the patient during transport ^(^ ^4^ ^,^ ^21^ ^-^ ^22^ ^,^ ^36^ ^,^ ^42^ ^)^ .
	To perform procedures of various complexities during the flight, according to the patient’s clinical condition ^(^ ^7^ ^,^ ^9^ ^,^ ^22^ ^-^ ^23^ ^,^ ^25^ ^,^ ^27^ ^-^ ^30^ ^,^ ^36^ ^,^ ^39^ ^,^ ^41^ ^,^ ^48^ ^-^ ^50^ ^)^ .
	To perform orotracheal intubation when necessary ^(^ ^51^ ^)^ .
**POST-FLIGHT**	To have good written communication skills to complete patient documentation in forms or a proprietary system, organize and protocol records chronologically ^(^ ^19^ ^,^ ^22^ ^,^ ^26^ ^,^ ^35^ ^,^ ^40^ ^)^ .
	To provide clear technical communication by providing a detailed description of the patient’s case to the ground health team, indicating all changes and interventions performed during pre-flight and during the flight ^(^ ^19^ ^,^ ^22^ ^,^ ^26^ ^,^ ^40^ ^)^ .
	To disinfect health equipment and replenish materials for the next mission ^(^ ^22^ ^)^ .

## Discussion

 This scoping review enabled mapping the competencies of aerospace nurses in the context of disasters, which were systematically presented in three phases: pre-flight (12 competencies), flight (11 competencies), and post-flight (three competencies), aimed at guiding professionals, managers, educators, and researchers ^(^ ^4^ ^,^ ^7^ ^,^ ^9^ ^,^ ^18^ ^-^ ^51^ ^)^ . 

 It became evident that developing these competencies requires a variety of specific knowledge, skills, and attitudes for care and management in scenarios with multiple and diverse challenges and levels of complexity, threats, vulnerabilities, and risks ^(^ ^4^ ^,^ ^7^ ^,^ ^9^ ^,^ ^18^ ^-^ ^51^ ^)^ . 

 During disasters, the use of aircraft for rescue and transportation of patients of different models and sizes, with both rotary-wing and fixed-wing, may be necessary. In this sense, the review demonstrated that the aerospace nurse plays a strategic role within the flight team in deciding which type of aircraft is most suitable for each situation ^(^ ^18^ ^-^ ^19^ ^,^ ^36^ ^,^ ^44^ ^)^ . 

 It is the responsibility of the aerospace nurse to assess, alongside the flight team, the conditions of the rescue site, the severity of the event, the number of victims to be transported, the individual needs of each patient, and the elements involved to enable the victim transportation and rescue while maintaining the safety and integrity of both the victims and the team aboard the aircraft ^(^ ^18^ ^-^ ^19^ ^,^ ^36^ ^,^ ^44^ ^)^ . 

 In general, rotary-wing aircraft cover short distances, have better access to the patients’ original location, and often allow transportation at low altitudes, unlike fixed-wing aircraft, which cover long distances, transport a larger number of non-infectious victims, and can reach higher altitudes ^(^ ^9^ ^,^ ^18^ ^,^ ^44^ ^)^ . 

 It was evident that the aerospace nurse must have specific knowledge of flight physiology to predict and immediately identify any risks or changes in the patient’s clinical condition due to the hypobaric environment For example, altitude hypoxia, the effects of cabin pressurization, clinical pharmacokinetics and pharmacodynamics in a flight environment, and changes in equipment patterns ^(^ ^7^ ^,^ ^9^ ^,^ ^19^ ^,^ ^34^ ^)^ . 

 Furthermore, knowledge of flight safety procedures and aeronautical emergencies is necessary to mitigate situations arising from aircraft speed, noise, vibrations, and gravitational forces. It is important to develop knowledge about the correct positioning of patients, perceive the risk of displacement of materials and equipment that may occur during takeoff and landing, and potentially cause accidents ^(^ ^7^ ^,^ ^9^ ^,^ ^19^ ^,^ ^34^ ^)^ . 

 In air transport and rescue, it is essential for the nurse to possess technical skills that encompass well-founded knowledge of disaster phenomena and their different typologies (wars, terrorist attacks, floods, landslides, heat waves, cold waves, epidemics, pests, accidents involving CBRN materials, earthquakes, tsunamis, among others), as well as the ability to deal with multiple victims, which is a common situation in such events ^(^ ^9^ ^,^ ^19^ ^,^ ^24^ ^,^ ^27^ ^,^ ^34^ ^)^ . Indeed, by understanding the context, magnitude, and particularities of disasters, the aerospace nurse will be better equipped to prepare the aircraft and equipment for the mission. 

 To successfully carry out this activity, the use of checklists for materials, supplies, medications, and other necessary supplies is necessary, which are standardized for the aircraft, the type of disaster, as well as for the complexity and expected duration of the mission. These items are essential to ensure that patient care during the flight is carried out without incidents, in order to avoid the lack of any material or equipment and to maintain adequate conditions of use and operation of materials and equipment. Thus, it is ensured that procedures will be performed correctly, maximizing the potential benefit to patients ^(^ ^7^ ^,^ ^9^ ^,^ ^22^ ^-^ ^23^ ^,^ ^25^ ^,^ ^27^ ^,^ ^29^ ^-^ ^30^ ^,^ ^49^ ^-^ ^50^ ^)^ . 

 The studies highlighted the need for planning for air transportation, aerial rescue, and care starting from the establishment of the Nursing Process, supported by Nursing theories ^(^ ^52^ ^-^ ^53^ ^)^ . They also pointed out the need for creativity and the use of instruments and technologies, such as applications and artificial intelligence, to promote, in a short time and in high complexity scenarios, the medical history and physical examination of the patient, the establishment of priority Nursing diagnoses, and essential interventions to be executed and adequately maintained before, during, and after the flight, with the purpose of overcoming the challenges arising from the aerial environment ^(^ ^7^ ^,^ ^18^ ^-^ ^19^ ^,^ ^23^ ^,^ ^26^ ^,^ ^33^ ^,^ ^43^ ^)^ . 

 Technical skills, along with communication abilities to deal with multiple victims, are highlighted in various studies ^(^ ^7^ ^,^ ^9^ ^,^ ^22^ ^-^ ^23^ ^,^ ^25^ ^,^ ^27^ ^-^ ^28^ ^,^ ^30^ ^,^ ^46^ ^,^ ^49^ ^)^ . Also widely emphasized is the constant monitoring of patients, which includes pulse oximetry and oxygen administration to prevent altitude hypoxia when necessary ^(^ ^7^ ^,^ ^9^ ^,^ ^22^ ^-^ ^23^ ^,^ ^25^ ^,^ ^27^ ^-^ ^28^ ^,^ ^30^ ^,^ ^40^ ^,^ ^46^ ^)^ . Other essential procedures include attention to breathing patterns, observation of signs of pneumothorax, and maintenance of chest drains in full working order. Additionally, administering medications, preparing for situations of psychomotor agitation, and preventing seizures are crucial ^(^ ^9^ ^,^ ^19^ ^,^ ^22^ ^,^ ^26^ ^-^ ^28^ ^,^ ^30^ ^,^ ^32^ ^,^ ^42^ ^-^ ^43^ ^)^ . 

 It is noteworthy that the aerospace nurse often performs technical procedures during the pre-flight phase to avoid carrying them out in a noisy environment with vibrations and reduced lighting produced during flight. However, when necessary, procedures must be performed during the flight, with extra precautions to ensure the safety of the patient, the professional, and the aircraft ^(^ ^9^ ^,^ ^19^ ^,^ ^22^ ^,^ ^26^ ^-^ ^28^ ^,^ ^30^ ^,^ ^32^ ^,^ ^42^ ^-^ ^43^ ^)^ . 

 The sources demonstrated the nurse’s involvement in orotracheal intubation in the aerospace context, when necessary ^(^ ^51^ ^)^ . Indeed, such practice has been recorded in the USA, where legislation supports nurses in performing this type of procedure ^(^ ^54^ ^)^ . 

 The sources also emphasize skills related to leadership, effective communication, safety practices, team organization, and resource management during the flight ^(^ ^9^ ^,^ ^19^ ^,^ ^24^ ^,^ ^27^ ^,^ ^34^ ^)^ . In disasters, the aerospace nurse needs to develop skills focused on team management to ensure an effective and rapid response ^(^ ^9^ ^,^ ^19^ ^,^ ^24^ ^,^ ^27^ ^,^ ^34^ ^)^ . The ability to make well-founded and assertive decisions is crucial for dealing with unpredictable situations. Furthermore, in environments with limited resources, knowing how to manage materials, equipment, and personnel is vital to ensure that all available resources are used optimally and remain operational ^(^ ^28^ ^,^ ^45^ ^)^ . 

 In general, disaster phenomena can cause various types of damage to patients, including multiple injuries, bruises, fractures, lacerations, amputations, hemorrhages, firearm injuries, intoxications, respiratory injuries, infections, hyper/hypothermia, burns, dehydration, exhaustion, as well as acute stress and post-traumatic stress disorder ^(^ ^35^ ^)^ . It is the responsibility of the aerospace nurse to be prepared to deal with the particularities of these occurrences, ensuring that the patient receives the necessary nursing interventions until reaching the destination hospital ^(^ ^35^ ^)^ . 

 Furthermore, patient safety is a significantly addressed issue in the context of aerospace nursing. Therefore, the nurse needs to recognize safety practices related to aircraft on the ground or in flight in order to intervene, promoting and providing safety ^(^ ^9^ ^,^ ^37^ ^,^ ^46^ ^,^ ^47^ ^-^ ^48^ ^)^ . 

 In the academic field, it is highlighted that the pursuit of excellence in professional education, both in undergraduate and postgraduate studies, and in courses for the training of military nurses, has the potential to enhance safety during flights, ensure the protection of the transported team, optimize the use of time and necessary resources, and above all, offer high-quality, sustainable, and appropriate assistance for the aerial transportation of patients ^(^ ^7^ ^,^ ^45^ ^)^ . 

 In addition, knowledge about flight safety and possible incidents on aircraft helps ensure the safety of the aerospace nurse and other flight team members, such as in cases of cabin depressurization and severe turbulence. This includes knowing how to act in these emergency situations, how to use the prescribed safety equipment, and following emergency evacuation protocols if necessary, which tends to allow for more effective collaboration with the air crew ^(^ ^9^ ^,^ ^37^ ^,^ ^45^ ^,^ ^47^ ^-^ ^48^ ^)^ . 

 These findings reveal the diversity of competencies required of aerospace nurses in critical situations, demonstrating the need for a broad set of technical knowledge, communication skills, management, and leadership to ensure high-quality care and safety during the planning of aerial patient transport, which commonly becomes more complex in disaster contexts ^(^ ^9^ ^,^ ^19^ ^,^ ^24^ ^,^ ^27^ ^,^ ^34^ ^)^ . 

 During rescues, many of the injured individuals experience physical and psychosocial discomforts. Pain, nausea, and anxiety/distress management fall within the purview of aerospace nurses, as does the promotion of patient comfort, which is an effective tool for mitigating or controlling the effects of these clinical occurrences ^(^ ^29^ ^)^ . 

 In the post-flight phase, competencies emphasized the importance of written communication, proper documentation, and equipment maintenance ^(^ ^19^ ^,^ ^22^ ^,^ ^26^ ^,^ ^35^ ^)^ . These aspects highlight the need for a meticulous and organized approach in this phase, ensuring not only accurate and clear documentation but also care for equipment and materials to guarantee the continuity and safety of the service in future aerial patient transport operations ^(^ ^22^ ^)^ . 

 At the end of the rescue, nursing documentation procedures continue. Therefore, the aerospace nurse must possess adequate knowledge and oratory skills to ensure good communication, both technically and clearly, in describing patient cases and in providing a chronological indication of all changes and procedures/care performed during the flight ^(^ ^19^ ^,^ ^22^ ^,^ ^24^ ^,^ ^35^ ^)^ . At the end, the aerospace nurse disinfects the equipment, requests and replaces the necessary materials for the next mission ^(^ ^22^ ^)^ . 

 Based on the findings, it is evident that the breadth and complexity of these competencies (before, during, and after flight) necessitate the development of innovations and technologies in care, education, and management. These can be systematically employed in the preparation processes for nurses, aiming to enhance the operationalization of both care and managerial actions ^(^ ^19^ ^,^ ^22^ ^,^ ^24^ ^,^ ^35^ ^)^ . 

 In disaster situations, there is a tendency for occurrences in the three phases of aerospace nursing care to be improperly recorded due to the chaos. However, the exercise of this competency is essential during the nursing history, as this information is crucial for clinical judgment, identifying diagnoses, and implementing nursing interventions. It also aids in the decision-making process regarding whether or not to transport the patient by air ^(^ ^20^ ^,^ ^25^ ^,^ ^38^ ^)^ . Additionally, it is vital to relay any in-flight incidents to ground teams ^(^ ^19^ ^,^ ^22^ ^,^ ^26^ ^,^ ^35^ ^)^ . 

 The scientific production in the field of aerospace nursing has shown that some countries, such as the United States, have standardized various activities for nurses. In Brazil, this area is still expanding and remains heavily influenced by the military model. There is a need for greater dissemination, legalization, and legitimization of these activities, especially considering the dynamic and complex contexts of disasters. These contexts require the implementation of measures guided by scientific evidence and knowledge grounded in effective and systematic (inter)disciplinary and intersectoral practices ^(^ ^4^ ^,^ ^7^ ^,^ ^9^ ^,^ ^18^ ^,^ ^21^ ^-^ ^23^ ^,^ ^34^ ^-^ ^37^ ^)^ . 

 Notably, the specialty of aerospace nursing was recognized in Brazil by the resolution of the Federal Nursing Council (COFEN) No. 581/2018 (amended by resolution COFEN No. 264/2023) ^(^ ^55^ ^)^ . Thus, through this legal provision, it is a specialty that lacks legal advancements and recognition in the civilian field in the Brazilian context, highlighting the need for more scientific investigations to consolidate its foundations 

It is noted that this review presented limitations due to the difficulty in retrieving some publications, which was partially circumvented through other search methods, such as direct contact with authors via email and university libraries, allowing access to 15 of the 37 articles in full. Concerning other search methods, no new articles were included as they did not address the competencies of aerospace nursing in the context of disasters. In this case, 12 publications had already been previously included in the study.

Given the inherent complexities of these situations, the grouping of identified competencies reveals the importance of mastering flight physiology, aeronautical emergency procedures, communication skills, leadership, and responsibilities in preparation, as well as actions during and after the flight. This mapping not only contributes to a clearer understanding of the role of the aerospace nurse but also to the development of effective strategies for training, skill development, and crisis management in this challenging context. These strategies should be oriented by competencies and the specific types of disasters.

## Conclusion

The mapping of the competencies of aerospace nurses in disaster scenarios has led to a broader understanding of the technical skills, specific knowledge, and attitudes required to enhance effective and safe assistance during the pre-flight, in-flight, and post-flight phases.

Given the demands faced by aerospace nurses in disaster situations, it is essential to develop a comprehensive set of competencies to ensure high-quality care for patients throughout all phases of air transport. The emphasis is on the urgent need to assess the necessity of using aircraft, which requires technical skills, knowledge of flight physiology, and aeronautical emergency procedures. In summary, the role of aerospace nurses in disaster scenarios during air transport requires a combination of technical competencies involving care, leadership, communication, and management. This highlights the ongoing need for training and updates to ensure excellence in care and the safety of both patients and the team involved in these challenging situations. This implies the need to consider the development of technologies for care, education, and management, as well as regulatory frameworks and legal provisions to provide legal support for these professionals committed to saving and maintaining lives. Future studies are suggested to validate these competencies among aerospace nurses and researchers in disaster contexts, in order to enhance the response capacity to these phenomena.
