# Supplementary material for: Aerospace nurses’ competencies in disaster situations: a scoping review
Source: Rev Lat Am Enfermagem. 2024 Sep 23;32:e4326. doi: 10.1590/1518-8345.7421.4326 (PMC11421517; doi:10.1590/1518-8345.7421.4326)
Supplement: Translation

# Translation: Competencias del enfermero aeroespacial en situaciones de desastre: revisión de alcance

**DOI:** 10.1590/1518-8345.7421.4325

**Authors:** Bernardo Arnulpho Coelho de Paula, Débora Fernanda Haberland, Fábio José de Almeida Guilherme, Bruno Leal Barbosa, Alexandre Barbosa de Oliveira, Thiago Augusto Soares Monteiro da Silva

**Affiliations:** ^1^ Universidade de Vassouras, Curso de Enfermagem, Vassouras, RJ, Brasil.; ^2^ Universidade Federal do Rio de Janeiro, Escola de Enfermagem Anna Nery, Rio de Janeiro, RJ, Brasil.; ^3^ Força Aérea Brasileira, Instituto de Medicina Aeroespacial Brigadeiro Médico Roberto Teixeira, Rio de Janeiro, RJ, Brasil.

**Keywords:** Enfermería, Ambulancias Aéreas, Ciencia del Desastre, Medicina Aeroespacial, Urgencias Médicas, Equipos Humanos

## Abstract

**Objetivo:**

mapear las competencias del enfermero aeroespacial en situaciones de desastre.

**Método:**

revisión de alcance que siguió los pasos recomendados por el JBI y la *checklist Preferred Reporting Items for Systematic reviews and Meta-Analyses extension for Scoping Reviews* (PRISMA-ScR). Fue realizada en tres fases por dos revisores independientes, con cegamiento y con el apoyo de un tercer revisor para resolver desacuerdos. La selección se basó en el análisis de títulos, descriptores y resúmenes, con criterios de elegibilidad específicos, seguida de la lectura completa de los textos. Al final de la fase de selección se incluyeron 37 publicaciones.

**Resultados:**

los resultados indicaron que es necesario desarrollar habilidades técnicas, tener conocimiento sobre fisiología de vuelo, estar familiarizado con los procedimientos de emergencia aeronáuticos, tener habilidades de comunicación, liderazgo y responsabilidades en la preparación de aeronaves. Durante el transporte, los enfermeros realizan anamnesis, exámenes físicos, monitoreo de pacientes, procedimientos clínicos y gestionan las complicaciones. Después del vuelo, realizan registros, procedimientos, higienizan el equipamiento clínico y reponen los insumos.

**Conclusión:**

debido a la complejidad de las prácticas de enfermería aeroespacial en situaciones de desastre, es necesario que los profesionales desarrollen competencias para garantizar una atención segura y eficaz. Hay que desarrollar tecnologías, marcos regulatorios y disposiciones legales que les brinden apoyo legal, al igual que futuros estudios para validar y profundizar las competencias mapeadas.

## Destacados

**(1)** Es necesario tener habilidades técnicas y conocimiento sobre fisiología de vuelo.

**(2)** Se destaca lo compleja que es la enfermería aeroespacial en desastres.

**(3)** Urgencia en la evaluación de responsabilidades en la preparación de aeronaves.

**(4)** Es necesario estar familiarizado con los procedimientos de emergencia aeronáuticos.

## Introducción

 Los desastres son fenómenos provocados por eventos adversos, que, en general, afectan a un ecosistema susceptible, causando daños a los seres humanos, a los bienes materiales y al medio ambiente ^(^ ^1^ ^)^ . Independientemente de si los eventos son de origen natural, tecnológico o social, los riesgos de los mismos aumentan cuando hay seres humanos expuestos y cuando se asocian con vulnerabilidades y la capacidad de preparación y respuesta se ve comprometida ^(^ ^1^ ^)^ . En esas situaciones, es común solicitar aeronaves que mejoren el acceso y la velocidad de respuesta de atención para la salud y ayuda humanitaria para las comunidades afectadas ^(^ ^2^ ^)^ . 

 El servicio de rescate y evacuación aéreo se inició durante la guerra franco-prusiana (1870) y utilizaba globos no dirigibles ^(^ ^3^ ^)^ . Posteriormente, la invención de los aviones permitió que esa práctica evolucionara durante la Primera Guerra Mundial (1914-1918), aunque de forma rudimentaria, dado que los pacientes eran transportados en compartimentos ubicados delante del piloto y sin la supervisión de los profesionales de la salud. El objetivo era el traslado rápido a un lugar seguro, donde se le pudiera brindar asistencia ^(^ ^3^ ^)^ . 

 En la Segunda Guerra Mundial (1939-1945), la Enfermería norteamericana logró avances significativos, ya que los heridos comenzaron a ser transportados en aviones de carga que contaban con tres camas cada uno y con la atención de “ *flight nurses* ” ( *flight Nightingales* ) ^(^ ^4^ ^-^ ^6^ ^)^ . Posteriormente, en Brasil, el Cuerpo de Bomberos Militares del Estado de Río de Janeiro comenzó a sistematizar el traslado aeromédico, y las actividades de rescate y salvamento comenzaron en 1988 ^(^ ^3^ ^-^ ^4^ ^)^ . 

 En la práctica, este tipo de transporte de pacientes consiste en el uso de diferentes aeronaves, de alas fijas (aviones) o de alas giratorias (helicópteros), que comúnmente se utilizan para realizar un transporte más rápido, cuando se recorren distancias cortas o en zonas de difícil acceso ^(^ ^7^ ^)^ . 

 En contextos más complejos, como las emergencias de salud pública y los desastres, ya sean de origen natural, tecnológico o social, el transporte aéreo para trasladar pacientes busca salvar la mayor cantidad de vidas posible, además de brindarle asistencia a los hospitales que deben atender un gran número de víctimas ^(^ ^8^ ^)^ . 

 Por lo tanto, es necesario capacitar/preparar a los enfermeros para este tipo de actividad, dado que generalmente forman parte importante de los equipos de salud del transporte aeroespacial ^(^ ^9^ ^-^ ^10^ ^)^ . Hay que destacar el papel que juegan los enfermeros en las situaciones de desastre que, además de brindar asistencia directa, desempeñan funciones de gestión dirigidas a reducir potenciales riesgos y problemas de salud que puedan afectar a las víctimas y al equipo ^(^ ^11^ ^)^ . 

 Se han realizado estudios a escala global sobre las competencias del enfermero en situaciones de desastre ^(^ ^2^ ^,^ ^8^ ^-^ ^12^ ^)^ . Este movimiento ha demostrado que es necesario ampliar las discusiones sobre el tema e incorporar evidencia sobre el desarrollo de las competencias específicas que se requieren en diferentes tipos de desastres. Esto implica pensar en realizar una revisión continua y mejorar las estrategias de capacitación, con el fin de garantizar mayor operatividad en las misiones y un patrón de respuesta más asertivo, sistematizado y orientado por acciones de cuidado y gestión basadas en la ciencia de la Enfermería ^(^ ^9^ ^,^ ^13^ ^)^ . 

 Por ende, la realización del presente estudio obedece a la necesidad de explorar este campo emergente y en constante evolución, para garantizar una mayor seguridad y eficacia en la asistencia, mejorar los programas de formación, desarrollar prácticas y políticas, e incluso que se realicen futuras investigaciones. Como punto de partida, se realizó una búsqueda preliminar en diciembre de 2023 en MEDLINE (a través de PubMed), *JBI Evidence Synthesis* , PROSPERO y *Cochrane Database of Systematic Reviews* , y no se encontraron otras revisiones sistemáticas o de alcance en curso o publicadas sobre las competencias del enfermero aeroespacial en situaciones de desastre. 

Por lo tanto, se planteó el siguiente objetivo: mapear las competencias del enfermero aeroespacial en situaciones de desastre.

## Método

### Tipo de estudio

 Se trata de una revisión de alcance realizada de acuerdo con las recomendaciones del JBI ^(^ ^14^ ^)^ , cuyo protocolo de investigación fue registrado en el *Open Science Framework* : osf.io/rh2t6 y DOI: 10.17605/OSF.IO/BRY5Q. 

 La pregunta de revisión fue: ¿cuáles son las competencias del enfermero aeroespacial en contextos de desastres? Se definió el nemotécnico PCC (P – Población, C – Concepto y C – Contexto): Población - enfermeros profesionales que se desempeñan en el escenario aeroespacial; Concepto - competencias, que considera la movilización de conocimientos, habilidades y actitudes necesarias para realizar actividades o funciones específicas ^(^ ^12^ ^)^ ; y Contexto - transporte aéreo en situaciones de desastre de cualquier tipo (naturales, tecnológicos o sociales). 

 A través de estos elementos se utilizaron los vocabularios controlados: Descriptores de Ciencias de la Salud (DeCS), *Medical Subject Headings* (MeSH) y Emtree ( *Embase Subject Headings* ). Después de realizar la búsqueda preliminar, se agregaron términos adicionales identificados en los títulos, resúmenes y descriptores/MESH de los artículos ( Figura 1 ). 

### Criterios de elegibilidad

Los criterios de elegibilidad fueron fuentes de información técnico-científica que abordaran las competencias del enfermero aeroespacial en situaciones de desastre, de acuerdo con el mnemónico PCC y la pregunta de revisión.

Se incluyeron estudios que estuvieran disponibles de forma completa, sin delimitación temporal o lingüística, ni de acceso abierto de fuentes, con el objetivo de ampliar el alcance de la investigación.

Se consideraron publicaciones de cualquier naturaleza, derivadas de estudios con enfoque cualitativo, cuantitativo y mixto. Por ende, se consideraron estudios primarios, diseños experimentales y cuasiexperimentales, revisiones, antes y después, observacionales, series de tiempo, estudios de cohorte y transversales, así como también casos y controles.

 También se consideraron estudios de la literatura gris (base de tesis y disertaciones, guías, protocolos, sitios *web* , opinión y *guidelines* ). Se excluyeron libros, capítulos y cartas al editor y publicaciones duplicadas. 

### Periodo

Las búsquedas se realizaron de octubre a diciembre de 2023.

### Fuentes de información

 Las búsquedas se realizaron en el Portal Regional de la Biblioteca Virtual en Salud (BVS), bajo la responsabilidad del Centro Latinoamericano y del Caribe de Información en Ciencias de la Salud (BIREME), que contiene bases de datos como: Índice Bibliográfico Español en Ciencias de la Salud (IBECS), Bases de Datos de Enfermería (BDENF), Literatura Latinoamericana y del Caribe en Ciencias de la Salud (LILACS), Red Peruana de Bibliotecas en Salud (LIPECS), *Scientific Electronic Library Online* (SciELO), *Medical Literature Analysis and Retrieval System Online* a través de PubMed, de la *National Library of Medicine* (NLM), entre otras. 

 Por medio del Portal de Revistas CAPES se accedió a las siguientes bases de datos: Ebsco: *Cumulative Index to Nursing and Allied Health Literature* (CINAHL) y *Academic Search Premier* (ASP); Elsevier: Embase y Scopus; *Clarivate Analytics* : *Web of Science* ; y, también, CAB *Direct* (plataforma que permite buscar en CAB *Abstracts* ) y *Global Health* . 

 La investigación también incluyó sitios *web* sobre enfermería aeroespacial, asistencia aeromédica, legislación profesional, el sitio *web* del *International Council of Nurses* (ICN), motores de búsqueda en internet y bibliotecas digitales que incluían tesis y disertaciones. Para sistematizar la búsqueda a través de la literatura gris se consideraron el portal integrador y de literatura gris *Science.gov* : USA.gov, *Epistemonikos: Database of the Best Evidence-Based Health Care* , *information Technologies and a network of experts* , *National Institute for Health and Care Excellence* (NICE). 

### Estrategias de búsqueda

Las búsquedas fueron realizadas en tres etapas por dos revisores, de forma independiente. Se mantuvo en ambos el proceso de cegamiento. La investigación contó con el apoyo de un tercer revisor para resolver desacuerdos y de una bibliotecaria vinculada a una universidad pública federal para orientar y realizar el seguimiento del proceso.

 En la primera etapa, se realizó el análisis de títulos, resúmenes y descriptores mediante una búsqueda inicial en el *Medical Literature Analysis and Retrieval System Online* (MEDLINE). Se utilizaron los operadores booleanos “AND” y “OR” junto con las palabras clave y los descriptores ( Figura 1 ). 

**Figura 1:** - Estrategias de búsqueda aplicadas en MEDLINE

**Investigación**	**Estrategias de Búsqueda**	**Resultados**
#01	**Search:** ”Nursing”[mh] OR Nursing ^*^ [tiab] OR “Nurses”[mh] ORNurse ^*^ [tiab] Sort by: Most Recent	690,130
#02	**Search:** ”Air Ambulances”[mh] OR Air Ambulance ^*^ [tiab] OR Emergency Helicopter ^*^ [tiab] OR Helicopter Ambulance ^*^ [tiab] OR”Aircraft”[mh] OR Aircraft ^*^ [tiab] OR Helicopter ^*^ [tiab] OR Airplane ^*^ [tiab] OR “Transportation of Patients”[mh] OR Patients Transportation ^*^ [tiab] OR Aeromedical evacuation[tiab] OR Airport ^*^ [tiab] OR “Aerospace Medicine”[mh] OR “Aviation Medicine”[tiab] OR “Space Medicine”[tiab] OR aerospace[tiab] OR Air[tiab] OR helicopter ^*^ [tiab] OR aircraft ^*^ [tiab] OR airplane ^*^ [tiab] Sort by: Most Recent	362,543
#03	**Search:** ”Disasters”[mh] OR Disaster ^*^ [tiab] OR Emergencies[mh] OR Emergenc ^*^ [tiab] OR Biological Disaster ^*^ [tiab] OR “Mass Casualty Incidents”[mh] OR Mass Casualty Incident ^*^ [tiab] OR Mass Casualt ^*^ [tiab] OR terror[tiab] OR Bioterrorism[tiab] OR Terrorism[tiab] Sort by: Most Recent	594,755
#04	**Search:** #01 AND #02 AND #03 Sort by: Most Recent	888

^*^ OR = o; ^†^ AND = y

La segunda etapa se desarrolló a partir de una búsqueda completa en todas las bases de datos y repositorios previamente seleccionados.

La tercera etapa se basó en el análisis de las listas de referencias de todas las fuentes que cumplían con los criterios de elegibilidad. Además, se buscó información adicional a través del contacto con los autores de los estudios primarios, incluso para obtener acceso a otros estudios sobre el tema.

### Selección de las fuentes

 Después de realizar las búsquedas, todas las citas identificadas se importaron a la aplicación Rayyan ^®^ ( *Qatar Computing Research Institute* , Doha, Qatar). Inicialmente, los datos fueron analizados mediante (re)lectura de los títulos, descriptores y resúmenes, siguiendo los criterios de elegibilidad. Los estudios excluidos se presentaron en el diagrama de flujo *Preferred Reporting Items for Systematic reviews and Meta-Analyses extension for Scoping Reviews* (PRISMA-ScR) ^(^ ^15^ ^)^ por población, concepto y contexto establecidos. 

 Se utilizó el administrador *web EndNote* ( *Clarivate* *Analytics* , PA, EE. UU.) para organizar las referencias e identificar estudios duplicados. 

Ambos investigadores leyeron los textos de forma completa, que fueron archivados en carpetas digitales. Los resultados obtenidos mediante la selección se presentaron en formato de diagrama de flujo, en base al modelo PRISMA-ScR.

### Extracción de datos

Después de realizar la lectura completa de los textos, se extrajeron los datos seleccionados con un instrumento adaptado, según las recomendaciones del JBI. Para operacionalizar el instrumento, se realizó una prueba piloto en tres fuentes, para que los revisores se familiarizaran con el proceso de selección, extracción y búsqueda de datos, en ese momento también se identificaron dudas y se realizaron algunos ajustes.

### Análisis y presentación de datos

 A partir de los datos extraídos se realizó un análisis de contenido inductivo ^(^ ^14^ ^)^ . La información contenía: título del artículo/documento, autores (nombre, título, profesión, institución, país), descriptores, tipo de fuente de información, idioma, objetivo(s), diseño y enfoque del estudio, origen y tipo de situación de desastre, competencias de enfermería aeroespacial en el contexto de desastres (en las fases antes del vuelo, durante el vuelo y después del vuelo). 

La síntesis de resultados se presentó en forma de flujo, tablas y diagrama de imágenes, según el objetivo y la pregunta de investigación.

## Resultados

 Se identificaron 4.981 publicaciones, de las cuales 1.882 estaban duplicadas. Por lo tanto, se importaron 3.099 publicaciones al *software* Rayyan. Después de la selección doble ciego por parte de los revisores, se excluyeron 2.765 fuentes. Por lo que quedaron para el análisis del texto completo 334 publicaciones. Luego del análisis pareado y ciego, se excluyeron 30 publicaciones por población, 152 por concepto y 18 por contexto. Se incluyeron 37 artículos ( Figura 2 ). 

 Utilizando otras estrategias de búsqueda, se recuperaron 796 publicaciones, 130 en *Google Scholar* y 666 mediante búsqueda inversa. De ese total, 12 estaban duplicadas y 784 fueron excluidas, 236 por población, 404 por contexto y 117 por concepto. Por lo tanto, no se incluyeron nuevas publicaciones a través de otras formas de búsqueda, dado que no se encontraron fuentes que abordaran el tema de las competencias del enfermero aeroespacial en el contexto de desastres, de acuerdo con los criterios de elegibilidad. En total, se incluyeron 37 publicaciones para revisión ( Figura 2 ). 

 De los 37 estudios sobre las competencias del enfermero aeroespacial en el contexto de desastres, que comenzaron a publicarse desde 1991, se identificó un promedio de dos a tres artículos publicados por año. Cabe destacar que, en 2010, 2011, 2017 y 2020 se identificaron tres publicaciones por año; en 2003, 2005, 2007, 2008, 2012, 2013, 2021, 2022 y 2023 hubo dos publicaciones por año, como se muestra en la Figura 3 . 

En lo que respecta a las proporciones, dicho resultado puede estar relacionado con los avances que se han producido en el campo de la Enfermería sobre el uso de prácticas de cuidado en el ámbito de los sistemas de aviación, especialmente en lo que respecta al aumento de la frecuencia y complejidad de los desastres, a una percepción más clara del riesgo ante esos eventos, y a la necesidad de discutir abordajes de múltiples víctimas en casos clínico-quirúrgicos complejos, que requieren transporte y rescate aeromédico en dichas situaciones.

 Cabe destacar que con el surgimiento de la especialidad de enfermería aeroespacial se torna necesario llevar a cabo la divulgación científica del tema y, por ende, la sistematización de esa práctica. También hay que considerar el movimiento del ICN que comenzó con mayor énfasis en 2009 y busca identificar estrategias para formalizar las competencias de los enfermeros para trabajar en desastres ^(^ ^10^ ^,^ ^16^ ^-^ ^17^ ^)^ . 

**Figura 2:** - Diagrama de flujo PRISMA-ScR

 En cuanto al país, se identificó que 23 estudios se realizaron en los Estados Unidos (EE.UU.), 11 en Brasil, uno en China, uno en Finlandia y uno en Irlanda. En lo que respecta al idioma, 26 fueron escritos en inglés y 11 en portugués. Se destaca la inversión realizada en transporte y rescate aeromédico en el ámbito civil y militar en estos países ( Figura 3 ) 

 Se identificó que siete artículos fueron publicados en *Air Medical Journal* , seis en la *Revista Brasileira de Enfermagem* , tres en *Critical Care Nursing Clinics* , dos en *Military Medicine* y dos en la revista *Texto & Contexto – Enfermagem* ( Figura 3 ). 

**Figura 3:** - Caracterización de las publicaciones mapeadas

**N.º**	**Año**	**Tipo de desastre**	**Fuente de información**	**País**	**Idioma**
F1 ^(^ ^18^ ^)^	1991	No especificado	*Rev Bras Enferm*	Brasil	Portugués
F2 ^(^ ^19^ ^)^	1995	No especificado	*Air Medical Journal*	Estados Unidos	Inglés
F3 ^(^ ^20^ ^)^	1996	No especificado	*Air Medical Journal*	Estados Unidos	Inglés
F4 ^(^ ^21^ ^)^	1997	Guerra	*Rev da Escola de Enfermagem da USP*	Brasil	Portugués
F5 ^(^ ^22^ ^)^	1999	Guerra	*Acta Paul Enferm*	Brasil	Portugués
F6 ^(^ ^23^ ^)^	2003	No especificado	*Rev Bras Enferm*	Brasil	Portugués
F7 ^(^ ^24^ ^)^	2003	No especificado	*Critical Care Nursing Clinics*	Estados Unidos	Inglés
F8 ^(^ ^25^ ^)^	2005	No especificado	*Critical Care Medicine*	Estados Unidos	Inglés
F9 ^(^ ^26^ ^)^	2005	Tsunami	*Scandinavian Journal of Surgery*	Finlandia	Inglés
F10 ^(^ ^27^ ^)^	2007	No especificado	*AORN Journal*	Estados Unidos	Inglés
F11 ^(^ ^28^ ^)^	2007	Huracán Katrina	*Disaster Management & Response*	Estados Unidos	Inglés
F12 ^(^ ^29^ ^)^	2008	Tsunami	*Air Medical Journal*	Estados Unidos	Inglés
F13 ^(^ ^30^ ^)^	2008	Guerra	*Critical Care Nursing Clinics of North America*	Estados Unidos	Inglés
F14 ^(^ ^31^ ^)^	2010	No especificado	*Critical Care Nurse*	Estados Unidos	Inglés
F15 ^(^ ^32^ ^)^	2010	No especificado	*Journal of Trauma Nursing| JTN*	Estados Unidos	Inglés
F16 ^(^ ^33^ ^)^	2010	Guerra	*International Journal of Nursing Practice*	Estados Unidos	Inglés
F17 ^(^ ^34^ ^)^	2011	No especificado	*Rev Bras Enferm*	Brasil	Portugués
F18 ^(^ ^35^ ^)^	2011	No especificado	*Texto & Contexto-Enfermagem*	Brasil	Portugués
F19 ^(^ ^36^ ^)^	2011	No especificado	*Rev Bras Enferm*	Brasil	Portugués
F20 ^(^ ^37^ ^)^	2012	No especificado	*Rev Bras Enferm*	Brasil	Portugués
F21 ^(^ ^38^ ^)^	2012	Guerra	*Air Medical Journal*	Estados Unidos	Inglés
F22 ^(^ ^39^ ^)^	2013	No especificado	*Journal of Emergency Nursing*	Estados Unidos	Inglés
F23 ^(^ ^40^ ^)^	2013	Guerra	*Journal of Emergency Nursing*	Estados Unidos	Inglés
F24 ^(^ ^41^ ^)^	2015	Guerra	*Journal of Emergency Nursing*	Estados Unidos	Inglés
F25 ^(^ ^42^ ^)^	2017	No especificado	*International Emergency Nursing*	Estados Unidos	Inglés
F26 ^(^ ^4^ ^)^	2017	Guerra	*Esc Anna Nery – Revista de Enfermagem*	Brasil	Portugués
F27 ^(^ ^43^ ^)^	2017	No especificado	*Military Medicine*	Estados Unidos	Inglés
F28 ^(^ ^44^ ^)^	2018	QBRN ^*^	*Air Medical Journal*	Estados Unidos	Inglés
F29 ^(^ ^45^ ^)^	2019	Guerra y desastres naturales	*Nurs Outlook*	Estados Unidos	Inglés
F30 ^(^ ^46^ ^)^	2020	No especificado	*Military Medicine*	Estados Unidos	Inglés
F31 ^(^ ^47^ ^)^	2020	No especificado	*Nurse Leader*	Estados Unidos	Inglés
F32 ^(^ ^48^ ^)^	2020	No especificado	*Scandinavian Journal of Trauma, Resuscitation and Emergency Medicine country*	Irlanda	Inglés
F33 ^(^ ^49^ ^)^	2021	No especificado	*Air Medical Journal*	Estados Unidos	Inglés
F34 ^(^ ^9^ ^)^	2021	No especificado	*Nursing*	Brasil	Portugués
F35 ^(^ ^50^ ^)^	2022	No especificado	*Hong Kong Journal of Emergency Medicine*	China	Inglés
F36 ^(^ ^51^ ^)^	2023	No especificado	*Air Medical Journal*	Estados Unidos	Inglés
F37 ^(^ ^7^ ^)^	2023	QBRN ^*^	*Texto & Contexto-Enfermagem*	Brasil	Portugués

^*^ QBRN = Químicos, biológicos, radiológicos y nucleares

 En cuanto a la tipología de los desastres, se identificó que 22 artículos abordaron el contexto del desastre en forma general, sin especificar la tipología; nueve se referían a guerras, dos a eventos que involucraban agentes químicos, biológicos, radiológicos y nucleares (QBRN), dos a eventos geológicos (tsunami), uno a un evento meteorológico (huracán) y uno estaba relacionado con un evento de origen natural (sin definir la subtipología exacta) ( Figura 3 ). 

Esta pluralidad de tipos de eventos sugiere que es necesario estudiar más el tema en cuestión y entender que las especificidades de cada evento pueden implicar prácticas orientadas a situaciones clínicas específicas. Además, comprender los desafíos que se enfrentan y las lecciones que se aprenden en este tipo de eventos es estratégico para tomar acciones de gestión del riesgo de desastres, lo que implica pensar en los procesos, es decir, en las acciones que hay que realizar antes, durante y después del evento. También se puede ver que en los estudios mapeados hay un cierto énfasis en las situaciones de guerra y de eventos QBRN, lo que nos lleva a reflexionar sobre las preocupaciones actuales que generan este tipo de eventos.

 En definitiva, las competencias del enfermero aeroespacial en el contexto de desastres, que fueron mapeadas en esta revisión de alcance, se agruparon en tres categorías, a saber: antes del vuelo, durante el vuelo y después del vuelo ( Figura 4 ). 

**Figura 4:** - Competencias del enfermero aeroespacial en el contexto de desastres

**Fase de transporte**	**Mapeo de las competencias del enfermero aeroespacial en el contexto de desastres**
**ANTES DEL VUELO**	Conocer la fisiología de vuelo y los procedimientos de emergencia aeronáuticos para brindar una atención de enfermería de calidad ^(^ ^7^ ^,^ ^9^ ^,^ ^19^ ^,^ ^33^ ^,^ ^36^ ^-^ ^37^ ^)^ .
	Conocer las prácticas de seguridad de vuelo, del paciente y del profesional relacionadas con aeronaves en tierra o en vuelo ^(^ ^9^ ^,^ ^33^ ^,^ ^36^ ^-^ ^37^ ^,^ ^45^ ^-^ ^48^ ^)^ .
	Conocer las aeronaves utilizadas en el transporte aéreo y comprender el uso del equipamiento médico relacionado ^(^ ^9^ ^,^ ^19^ ^,^ ^24^ ^,^ ^27^ ^,^ ^29^ ^,^ ^33^ ^,^ ^50^ ^)^ .
	Saber preparar la aeronave para la misión ^(^ ^9^ ^,^ ^19^ ^,^ ^24^ ^,^ ^27^ ^,^ ^46^ ^,^ ^49^ ^-^ ^50^ ^)^ .
	Realizar la *checklist* de los materiales y medicamentos estandarizados para uso en la aeronave ^(^ ^9^ ^,^ ^19^ ^,^ ^24^ ^,^ ^27^ ^,^ ^49^ ^-^ ^50^ ^)^ .
	Proporcionar los materiales, equipos y oxígeno necesarios ^(^ ^9^ ^,^ ^19^ ^,^ ^24^ ^,^ ^27^ ^,^ ^49^ ^-^ ^50^ ^)^ .
	Poseer perfil de liderazgo y capacidad para la toma de decisiones ^(^ ^28^ ^,^ ^45^ ^)^ .
	Actuar como formador profesional desarrollando programas en equipo para garantizar que el equipo tenga habilidades, competencias y conocimientos actualizados ^(^ ^45^ ^)^ .
	Poseer habilidades técnicas para enfrentar desastres, habilidades de comunicación y capacidad para lidiar con múltiples víctimas ^(^ ^9^ ^,^ ^19^ ^,^ ^24^ ^,^ ^27^ ^,^ ^34^ ^)^ .
	Tener buenas habilidades de comunicación oral y escrita para discutir casos con el equipo y realizar los informes necesarios ^(^ ^7^ ^,^ ^35^ ^,^ ^37^ ^,^ ^45^ ^)^ .
	Comprobar que sea necesario el uso de la aeronave, identificando la naturaleza de la emergencia a través del estado, número de víctimas y lugar del hecho ^(^ ^9^ ^,^ ^18^ ^,^ ^36^ ^,^ ^44^ ^)^ .
	Planificar toda la misión junto con el equipo multidisciplinario ^(^ ^22^ ^,^ ^36^ ^,^ ^44^ ^,^ ^51^ ^)^ .
	Realizar la evaluación del paciente (Historia Clínica de Enfermería) participar en la decisión de trasladar o no al paciente por vía aérea ^(^ ^20^ ^,^ ^25^ ^,^ ^35^ ^,^ ^38^ ^)^ .
	Realizar procedimientos de diversa complejidad durante la preparación del paciente para el transporte, según el cuadro clínico ^(^ ^7^ ^,^ ^9^ ^,^ ^22^ ^-^ ^23^ ^,^ ^25^ ^,^ ^27^ ^-^ ^28^ ^,^ ^30^ ^,^ ^49^ ^-^ ^50^ ^)^ .
**DURANTE EL VUELO**	Tener conocimiento de los procedimientos de emergencia de la aeronave en la que se realiza el transporte ^(^ ^25^ ^-^ ^26^ ^)^ .
	Tener la capacidad de desarrollar y gestionar acciones del plan de respuesta a desastres en un entorno aéreo ^(^ ^9^ ^,^ ^27^ ^,^ ^39^ ^)^ .
	Tener buena comunicación escrita para realizar registros y notas de enfermería ^(^ ^21^ ^-^ ^22^ ^)^ .
	Tener la capacidad de liderar el equipo de atención durante el vuelo ^(^ ^22^ ^)^ .
	Gestionar el flujo de pacientes, recursos humanos, materiales, equipamiento y suministros durante el vuelo ^(^ ^9^ ^,^ ^19^ ^,^ ^22^ ^,^ ^26^ ^-^ ^28^ ^,^ ^31^ ^-^ ^32^ ^,^ ^42^ ^,^ ^44^ ^)^ .
	Tener la capacidad de gestionar complicaciones durante el vuelo ^(^ ^22^ ^,^ ^36^ ^)^ .
	Utilizar instrumentos que garanticen la seguridad y salud del profesional (EPP y EPC) ^(^ ^7^ ^,^ ^40^ ^)^ .
	Realizar anamnesis y examen físico dirigido, y establecer el proceso de Enfermería ^(^ ^20^ ^,^ ^25^ ^,^ ^36^ ^,^ ^38^ ^-^ ^39^ ^,^ ^41^ ^)^ .
	Brindarle apoyo psicoemocional al paciente durante el transporte ^(^ ^4^ ^,^ ^21^ ^-^ ^22^ ^,^ ^36^ ^,^ ^42^ ^)^ .
	Realizar procedimientos de diversa complejidad durante el vuelo, según la condición clínica del paciente ^(^ ^7^ ^,^ ^9^ ^,^ ^22^ ^-^ ^23^ ^,^ ^25^ ^,^ ^27^ ^-^ ^30^ ^,^ ^36^ ^,^ ^39^ ^,^ ^41^ ^,^ ^48^ ^-^ ^50^ ^)^ .
	Realizar intubación orotraqueal si es necesario ^(^ ^51^ ^)^ .
**DESPUÉS EL VUELO**	Tener buena comunicación escrita para completar la documentación del paciente en un formulario o sistema específico, organizar y archivar los registros según el orden cronológico de los eventos ^(^ ^19^ ^,^ ^22^ ^,^ ^26^ ^,^ ^35^ ^,^ ^40^ ^)^ .
	Desarrollar una comunicación técnica clara mediante la descripción detallada del caso del paciente al equipo de salud en tierra, indicando todos los cambios e intervenciones realizadas antes y durante el vuelo ^(^ ^19^ ^,^ ^22^ ^,^ ^26^ ^,^ ^40^ ^)^ .
	Desinfectar el equipamiento sanitario y reponer los materiales previstos para la próxima misión ^(^ ^22^ ^)^ .

## Discusión

 Esta revisión de alcance permitió mapear las competencias del enfermero aeroespacial en el contexto de desastres, que se presentaron de manera sistemática en tres fases: antes del vuelo (12 competencias), durante el vuelo (11 competencias) y después del vuelo (3 competencias), con el fin de formar profesionales, gestores, educadores e investigadores ^(^ ^4^ ^,^ ^7^ ^,^ ^9^ ^,^ ^18^ ^-^ ^51^ ^)^ . 

 Es evidente que el desarrollo de esas competencias requiere mucho conocimiento, habilidades y actitudes específicas para la asistencia y gestión del cuidado en escenarios con múltiples y diferentes desafíos y niveles de complejidad, amenazas, vulnerabilidades y riesgos ^(^ ^4^ ^,^ ^7^ ^,^ ^9^ ^,^ ^18^ ^-^ ^51^ ^)^ . 

 En caso de desastres, puede ser necesario el uso de aeronaves de diferentes modelos y tamaños, con alas giratorias y fijas, para el rescate y transporte de pacientes. Por ello, la revisión demostró que el enfermero aeroespacial tiene un papel estratégico, junto con el equipo de vuelo, en la toma de decisiones sobre qué tipo de aeronave es la más adecuada para cada situación ^(^ ^18^ ^-^ ^19^ ^,^ ^36^ ^,^ ^44^ ^)^ . 

 Es el enfermero aeroespacial el que debe evaluar, junto con el equipo de vuelo, las condiciones del lugar de rescate, la gravedad del evento, el número de víctimas que hay que transportar, las necesidades individuales de cada paciente y los elementos necesarios para realizar el transporte y rescate de víctimas manteniendo la seguridad e integridad de las víctimas y del equipo en la aeronave ^(^ ^18^ ^-^ ^19^ ^,^ ^36^ ^,^ ^44^ ^)^ . 

 En general, las aeronaves de alas giratorias viajan distancias cortas, tienen mejor acceso al lugar de origen del paciente y muchas veces permiten el transporte a baja altura, a diferencia de las aeronaves de alas fijas, que cubren largas distancias y transportan un mayor número de víctimas con enfermedades no transmisibles, además de alcanzar mayores altitudes ^(^ ^9^ ^,^ ^18^ ^,^ ^44^ ^)^ . 

 Está claro que el enfermero aeroespacial debe tener conocimientos específicos sobre fisiología de vuelo, para predecir e identificar de inmediato posibles riesgos o cambios en el estado clínico del paciente derivados del ambiente hipobárico. Como, por ejemplo, hipoxia de altitud, efectos de la presurización de la cabina, farmacocinética clínica y farmacodinamia en un entorno de vuelo y cambios en los estándares del equipamiento ^(^ ^7^ ^,^ ^9^ ^,^ ^19^ ^,^ ^34^ ^)^ . 

 Además, es necesario que conozca los procedimientos de seguridad de vuelo y emergencias aeronáuticas, a fin de reducir las situaciones derivadas de la velocidad de las aeronaves, el ruido, la vibración y las fuerzas gravitacionales, adquiera conocimiento sobre el correcto posicionamiento del paciente, note el riesgo de desplazamiento de los materiales y del equipamiento que pueden ocurrir durante el despegue y el aterrizaje y, eventualmente, causar accidentes ^(^ ^7^ ^,^ ^9^ ^,^ ^19^ ^,^ ^34^ ^)^ . 

 Es fundamental que en el transporte aéreo y rescate los enfermeros cuenten con habilidades técnicas que abarquen conocimientos previos y fundamentados sobre los fenómenos de desastres y los diferentes tipos (guerras, ataques terroristas, inundaciones, deslizamientos de tierra, olas de calor y frío, epidemias, plagas, accidentes con materiales QBRN, terremotos, tsunamis, entre otros), además de la capacidad de atender múltiples víctimas, lo cual es habitual en este tipo de eventos ^(^ ^9^ ^,^ ^19^ ^,^ ^24^ ^,^ ^27^ ^,^ ^34^ ^)^ . De hecho, al comprender el contexto, la magnitud y las particularidades de los desastres, el enfermero aeroespacial podrá preparar mejor la aeronave y el equipamiento para la misión. 

 Para realizar esta actividad con éxito es necesario utilizar *checklists* de materiales, insumos, medicamentos y otros suministros necesarios, que están estandarizadas para la aeronave, el tipo de desastre y para la complejidad y el tiempo esperado de la misión. Estos elementos son esenciales para que la atención a los pacientes durante el vuelo se realice sin complicaciones, para evitar la falta de cualquier material o equipamiento y mantener adecuadas condiciones de uso y funcionamiento de los materiales y el equipamiento. Esto garantiza que los procedimientos se realicen correctamente y maximicen el beneficio potencial para los pacientes ^(^ ^7^ ^,^ ^9^ ^,^ ^22^ ^-^ ^23^ ^,^ ^25^ ^,^ ^27^ ^,^ ^29^ ^-^ ^30^ ^,^ ^49^ ^-^ ^50^ ^)^ . 

 Los estudios hicieron hincapié en que es necesario planificar el transporte, el rescate aéreo ^(^ ^18^ ^,^ ^41^ ^,^ ^47^ ^)^ y los cuidados mediante la implementación del Proceso de Enfermería, que se basa en las teorías de Enfermería ^(^ ^52^ ^-^ ^53^ ^)^ . También indicaron que era necesario tener creatividad para usar instrumentos y tecnologías, como aplicaciones e inteligencia artificial, para promover, en poco tiempo y en escenarios de alta complejidad ^(^ ^53^ ^)^ , la anamnesis y el examen físico del paciente, y determinar los Diagnósticos de enfermería prioritarios y las intervenciones esenciales que hay realizar y mantener adecuadamente antes, durante y después del vuelo, con el objetivo de superar los desafíos derivados del entorno aéreo ^(^ ^7^ ^,^ ^18^ ^-^ ^19^ ^,^ ^23^ ^,^ ^26^ ^,^ ^33^ ^,^ ^43^ ^)^ . 

 Varios estudios destacan las habilidades técnicas y las capacidades de comunicación que hay que tener para lidiar con múltiples víctimas ^(^ ^7^ ^,^ ^9^ ^,^ ^22^ ^-^ ^23^ ^,^ ^25^ ^,^ ^27^ ^-^ ^28^ ^,^ ^30^ ^,^ ^46^ ^,^ ^49^ ^)^ . También se hizo mucho hincapié, debido a la variedad de procedimientos que deben realizar los enfermeros, en el monitoreo constante de los pacientes, que incluye la oximetría de pulso y la administración de oxígeno para prevenir la hipoxia de altura en caso de ser necesario ^(^ ^7^ ^,^ ^9^ ^,^ ^22^ ^-^ ^23^ ^,^ ^25^ ^,^ ^27^ ^-^ ^28^ ^,^ ^30^ ^,^ ^40^ ^,^ ^46^ ^)^ . Otros procedimientos esenciales incluyen prestar atención al patrón de ventilación, observar signos de neumotórax y mantener los drenajes torácicos en pleno funcionamiento. Además de administración de medicamentos, preparación para situaciones de agitación psicomotora y prevención de crisis convulsivas ^(^ ^9^ ^,^ ^19^ ^,^ ^22^ ^,^ ^26^ ^-^ ^28^ ^,^ ^30^ ^,^ ^32^ ^,^ ^42^ ^-^ ^43^ ^)^ . 

 Cabe destacar que el enfermero aeroespacial, con frecuencia, realizará procedimientos técnicos durante la fase antes del vuelo, para evitar realizar procedimientos en un ambiente ruidoso, con vibraciones e iluminación reducida producto del vuelo. Sin embargo, si es necesario realizar procedimientos durante el vuelo, debe hacerse teniendo extrema precaución para preservar la seguridad del paciente, del profesional y de la aeronave ^(^ ^9^ ^,^ ^19^ ^,^ ^22^ ^,^ ^26^ ^-^ ^28^ ^,^ ^30^ ^,^ ^32^ ^,^ ^42^ ^-^ ^43^ ^)^ . 

 Las fuentes demostraron el papel del enfermero en las intubaciones orotraqueales en el contexto aeroespacial, cuando es necesario ^(^ ^51^ ^)^ . De hecho, esta práctica fue registrada en EE.UU., donde la legislación habilita al enfermero a realizar este tipo de procedimientos ^(^ ^54^ ^)^ . 

 Las fuentes también insisten en las habilidades relacionadas con el liderazgo, la comunicación efectiva, las prácticas de seguridad, la organización de equipos y la gestión de recursos durante el vuelo ^(^ ^9^ ^,^ ^19^ ^,^ ^24^ ^,^ ^27^ ^,^ ^34^ ^)^ . En situaciones de desastre, el enfermero aeroespacial debe desarrollar habilidades de gestión de equipos para garantizar una respuesta eficaz y rápida ^(^ ^9^ ^,^ ^19^ ^,^ ^24^ ^,^ ^27^ ^,^ ^34^ ^)^ . La capacidad de tomar decisiones asertivas y bien fundamentadas es esencial para afrontar situaciones impredecibles. Además, en entornos con recursos limitados, saber gestionar los materiales, el equipamiento y el personal es vital para garantizar que todos los recursos disponibles se utilicen de forma óptima y permanezcan operativos ^(^ ^28^ ^,^ ^45^ ^)^ . 

 En general, los fenómenos de desastre les pueden causar daños de distinta índole a los pacientes, relacionados con múltiples lesiones, contusiones, fracturas, laceraciones, amputaciones, hemorragias, heridas por armas, intoxicaciones, lesiones respiratorias, infecciones, hiper/hipotermia, quemaduras, deshidratación, agotamiento, incluso estrés agudo y trastorno de estrés postraumático ^(^ ^35^ ^)^ . Le corresponde al enfermero aeroespacial estar preparado para afrontar las particularidades de esos eventos y garantizar que el paciente reciba las intervenciones de enfermería necesarias hasta llegar al hospital de destino ^(^ ^35^ ^)^ . 

 Además, la seguridad del paciente es una cuestión que se aborda bastante en el contexto de la enfermería aeroespacial. Por lo tanto, el enfermero debe (re)conocer las prácticas de seguridad relacionadas con las aeronaves en tierra o en vuelo, para poder intervenir, promoviendo y brindando seguridad ^(^ ^9^ ^,^ ^37^ ^,^ ^45^ ^,^ ^47^ ^-^ ^48^ ^)^ . 

 En el ámbito académico, se destaca que la búsqueda de la excelencia en la formación profesional, en las carreras de grado y posgrado *lato sensu* y en los cursos de formación de enfermeros militares, puede mejorar la seguridad durante el vuelo, garantizar la protección del equipo transportado, optimizar el uso del tiempo y los recursos necesarios y, sobre todo, brindar una atención de calidad, sostenible y adecuada para el transporte aéreo de pacientes ^(^ ^7^ ^,^ ^45^ ^)^ . 

 Además, el conocimiento sobre la seguridad del vuelo y las posibles complicaciones en la aeronave ayudan a garantizar la seguridad del enfermero aeroespacial y de otros miembros del equipo de vuelo, como en casos de despresurización de la cabina y turbulencias severas. Esto incluye saber cómo actuar en esos casos de emergencia, cómo utilizar el equipo de seguridad previsto y seguir los protocolos de evacuación de emergencia de la aeronave, si es necesario, lo que favorece una colaboración más efectiva con la tripulación aérea ^(^ ^9^ ^,^ ^37^ ^,^ ^45^ ^,^ ^47^ ^-^ ^48^ ^)^ . 

 Estos hallazgos revelan las diferentes competencias que debe tener el enfermero aeroespacial en situaciones críticas, lo que demuestra que es necesario contar con un amplio conjunto de conocimientos técnicos, habilidades de comunicación, gestión y liderazgo para garantizar una atención de alta calidad y seguridad durante la planificación del transporte aéreo de pacientes, que, en general, se torna más compleja en contextos de desastres ^(^ ^9^ ^,^ ^19^ ^,^ ^24^ ^,^ ^27^ ^,^ ^34^ ^)^ . 

 Durante los rescates, muchos de los heridos experimentan malestar físico y psicosocial. El enfermero aeroespacial es responsable del manejo del dolor, las náuseas y la ansiedad/angustia y de promover el confort del paciente, que es una herramienta eficaz para minimizar o controlar los efectos de los síntomas clínicos ^(^ ^29^ ^)^ . 

 En la fase después del vuelo, las competencias enfatizaron la importancia de la comunicación escrita, la documentación adecuada y el mantenimiento del equipamiento ^(^ ^19^ ^,^ ^22^ ^,^ ^26^ ^,^ ^35^ ^)^ . Dichos aspectos destacan que es necesario adoptar un abordaje minucioso y organizado en esta etapa, para asegurarse que la documentación sea precisa y clara, que se cuidaran el equipamiento y los materiales, para garantizar la continuidad y seguridad del servicio en futuras operaciones de transporte aéreo de pacientes ^(^ ^22^ ^)^ . 

 Cuando termina el rescate, continúan los trámites relacionados con el registro de Enfermería. Para ello, el enfermero aeroespacial debe tener conocimientos y habilidades oratorias adecuadas, con el fin de garantizar una buena comunicación, de manera técnica y clara, para describir los casos de los pacientes y para indicar de forma cronológica todos los cambios y procedimientos/cuidados realizados durante el vuelo ^(^ ^19^ ^,^ ^22^ ^,^ ^24^ ^,^ ^35^ ^)^ . Al finalizar, el enfermero aeroespacial desinfecta el equipamiento, solicita y repone los materiales necesarios para la próxima misión ^(^ ^22^ ^)^ . 

 A partir de los hallazgos, se observa que la amplitud y complejidad de esas competencias (antes, durante y después del vuelo) implican pensar en el desarrollo de innovaciones y tecnologías para la atención, la enseñanza y la gestión, que puedan ser utilizadas sistemáticamente en los procesos de preparación del enfermero, con el objetivo de mejorar la operacionalización de las acciones de atención y gestión ^(^ ^19^ ^,^ ^22^ ^,^ ^24^ ^,^ ^35^ ^)^ . 

 Se observa que, debido al caos que se genera en las situaciones de desastre, hay cierta tendencia a que los sucesos no se registren correctamente en las tres fases de la atención de enfermería aeroespacial. Sin embargo, se afirma que es necesario desarrollar esa competencia para elaborar la historia clínica de Enfermería, considerando que dicha información es crucial para el juicio clínico, la identificación de diagnósticos y la implementación de intervenciones de Enfermería, además contribuye a decidir si se traslada o no al paciente por vía aérea ^(^ ^20^ ^,^ ^25^ ^,^ ^38^ ^)^ , sin olvidar que es necesario informarles las complicaciones que ocurren durante el vuelo a los equipos en tierra ^(^ ^19^ ^,^ ^22^ ^,^ ^26^ ^,^ ^35^ ^)^ . 

 La producción científica en el área de enfermería aeroespacial ha demostrado que algunos países, como Estados Unidos, estandarizaron diferentes actividades del enfermero. En Brasil, es un área que aún está en expansión y muy influenciada por el modelo militar, y que necesita mayor difusión, legalización y legitimación de las actividades, especialmente si se consideran los contextos dinámicos y complejos de los desastres, que requieren que se tomen medidas basadas en la evidencia científica y conocimiento basado en prácticas (inter)disciplinarias e intersectoriales efectivas y sistematizadas ^(^ ^4^ ^,^ ^7^ ^,^ ^9^ ^,^ ^18^ ^,^ ^21^ ^-^ ^23^ ^,^ ^34^ ^-^ ^37^ ^)^ . 

 Cabe destacar que la especialidad de enfermería aeroespacial fue reconocida en Brasil por la resolución del Consejo Federal de Enfermería (COFEN) n.º 581/2018 (modificada por la resolución COFEN n.º 264/2023) ^(^ ^55^ ^)^ . Por lo tanto, a través de esta disposición legal, se trata de una especialidad que carece de avances jurídicos y reconocimiento en el ámbito civil en el contexto brasileño, lo que demuestra que es necesario que se realicen más investigaciones científicas a fin de consolidar las bases de la misma. 

Se considera una limitación de esta revisión la dificultad para acceder a algunas publicaciones, que fue parcialmente superada mediante otras formas de búsqueda, como el contacto directo con los autores por correo electrónico y con bibliotecas universitarias, que permitieron acceder a 15 de los 37 artículos completos. No se incluyeron artículos nuevos mediante las otras formas de búsqueda, debido a que los artículos analizados no abordaban las competencias de enfermería aeroespacial en el contexto de desastres. En este caso ya se habían incluido previamente en el estudio 12 publicaciones.

Dadas las complejidades inherentes a ese tipo de situaciones, el conjunto de competencias identificadas revela la importancia de dominar la fisiología de vuelo, los procedimientos de emergencia aeronáuticos, las competencias de comunicación, el liderazgo y las responsabilidades en la preparación y el desempeño durante y después del vuelo. Este mapeo no solo contribuye a comprender mejor el papel del enfermero aeroespacial, sino también a desarrollar estrategias efectivas de formación, capacitación y gestión de crisis en ese contexto desafiante orientadas por las competencias y los tipos de desastres.

## Conclusión

El mapeo de las competencias del enfermero aeroespacial en situaciones de desastre determinó la identificación y comprensión más amplia de las habilidades técnicas, los conocimientos específicos y las actitudes necesarias para mejorar la asistencia efectiva y segura en las fases antes, durante y después del vuelo.

Dadas las demandas que enfrenta el enfermero aeroespacial en situaciones de desastre, hay que comprender que es necesario que desarrolle un conjunto integral de competencias para garantizarles una atención de alta calidad a los pacientes durante todas las fases del transporte aéreo. Se hace hincapié en la urgencia de evaluar la necesidad de utilizar aeronaves, que requieren habilidades técnicas, conocimiento sobre fisiología de vuelo y procedimientos de emergencia aeronáuticos. En definitiva, el papel del enfermero aeroespacial en desastres, durante el transporte aéreo, requiere una combinación de competencias técnicas que involucran atención, liderazgo, comunicación y gestión, y es necesario que tenga capacitación continua y se actualice para garantizar la excelencia en la atención y la seguridad tanto del paciente como la del equipo involucrado en esas situaciones desafiantes. Ello implica pensar en el desarrollo de tecnologías de atención, enseñanza y gestión, así como también en marcos regulatorios y disposiciones legales que les brinden apoyo legal a los profesionales comprometidos con salvar/ mantener a las personas con vida. Se sugieren que en el futuro se realicen estudios para que los enfermeros aeroespaciales y los investigadores validen dichas competencias en el contexto de desastres, con el fin de mejorar la capacidad de respuesta a estos fenómenos.

# Translation: Competências do enfermeiro aeroespacial em situações de desastres: revisão de escopo

**DOI:** 10.1590/1518-8345.7421.4327

**Authors:** Bernardo Arnulpho Coelho de Paula, Débora Fernanda Haberland, Fábio José de Almeida Guilherme, Bruno Leal Barbosa, Alexandre Barbosa de Oliveira, Thiago Augusto Soares Monteiro da Silva

**Affiliations:** ^1^ Universidade de Vassouras, Curso de Enfermagem, Vassouras, RJ, Brasil.; ^2^ Universidade Federal do Rio de Janeiro, Escola de Enfermagem Anna Nery, Rio de Janeiro, RJ, Brasil.; ^3^ Força Aérea Brasileira, Instituto de Medicina Aeroespacial Brigadeiro Médico Roberto Teixeira, Rio de Janeiro, RJ, Brasil.

**Keywords:** Enfermagem, Resgate Aéreo, Ciência do Desastre, Medicina Aeroespacial, Emergências, Equipe de Desastre

## Abstract

**Objetivo:**

mapear as competências do enfermeiro aeroespacial em situações de desastres.

**Método:**

revisão de escopo que seguiu as etapas recomendadas pelo JBI e o *checklist Preferred Reporting Items for Systematic reviews and Meta-Analyses extension for Scoping Reviews* (PRISMA-ScR). Foi conduzida em três fases por dois revisores independentes, com cegamento, e suporte de um terceiro revisor para resolver dissidências. A seleção baseou-se na análise dos títulos, descritores e resumos, com critérios de elegibilidade específicos, seguida pela leitura integral dos textos. Ao final da fase de seleção foram incluídas 37 publicações.

**Resultados:**

os resultados destacaram a necessidade de desenvolvimento de habilidades técnicas, conhecimento de fisiologia do voo, familiaridade com procedimentos de emergências aeronáuticas, habilidades de comunicação, liderança e responsabilidades na preparação de aeronaves. Durante o transporte, os enfermeiros realizam anamnese, exame físico, monitorização de pacientes, procedimentos clínicos e gerenciam intercorrências. Após o voo, fazem registros, desenvolvem procedimentos, realizam a higienização de equipamentos clínicos e a reposição de materiais de consumo.

**Conclusão:**

diante da complexidade das práticas de enfermagem aeroespacial em desastres, é necessário que os profissionais desenvolvam competências para garantir uma assistência segura e eficaz. Há que se desenvolver tecnologias, marcos regulatórios e previsões legais para suporte jurídico, bem como estudos futuros para validação e aprofundamento das competências mapeadas.

## Destaques

**(1)** Necessidade de habilidades técnicas e conhecimento de fisiologia do voo.

**(2)** Enfatiza-se a complexidade da enfermagem aeroespacial em desastres.

**(3)** Urgência na avaliação das responsabilidades na preparação de aeronaves.

**(4)** É necessário ter familiaridade com procedimentos de emergências aeronáuticas.

## Introdução

 Os desastres são fenômenos que resultam de eventos adversos, que geralmente impactam um ecossistema suscetível, ocasionando danos para os seres humanos, bens materiais e meio ambiente ^(^ ^1^ ^)^ . Sejam de origem natural, tecnológica ou social, esses eventos têm os seus riscos amplificados a partir da exposição humana e da associação de vulnerabilidades e capacidade de preparação e resposta comprometida ^(^ ^1^ ^)^ . Em tais situações, é prática comum a demanda por aeronaves que auxiliam no acesso, na celeridade da assistência à saúde e nas ações de ajuda humanitária às comunidades afetadas ^(^ ^2^ ^)^ . 

 O serviço de resgate e remoção aéreo teve início durante a Guerra Franco-Prussiana (1870), por meio de balões não direcionáveis ^(^ ^3^ ^)^ . Sequencialmente, a invenção dos aviões possibilitou a evolução dessa prática durante a 1ª Guerra Mundial (1914-1918), ainda que de forma rudimentar, uma vez que os pacientes eram transportados em compartimentos à frente do piloto e sem acompanhamento de profissionais de saúde. O propósito era a remoção ágil para um lugar seguro, onde houvesse alguma possibilidade de assistência ^(^ ^3^ ^)^ . 

 Na 2ª Guerra Mundial (1939-1945), a Enfermagem norte-americana alcançou um avanço significativo, visto que os feridos passaram a ser transportados em aviões de carga com três leitos cada, recebendo cuidados das “ *flight nurses* ” ( *Flying Nightingales* ) ^(^ ^4^ ^-^ ^6^ ^)^ . Mais tarde, no Brasil, a remoção aeromédica passou a ser sistematizada pelo Corpo de Bombeiros Militar do estado do Rio de Janeiro, cujas atividades, voltadas para resgate e salvamento, iniciaram-se em 1988 ^(^ ^3^ ^-^ ^4^ ^)^ . 

 Na prática, esse tipo de transporte de pacientes consiste no uso de diferentes aeronaves, de asas fixas (aviões) ou rotativas (helicópteros), sendo estas últimas comumente empregadas para transporte mais rápido, na ocasião de distâncias curtas ou áreas de difícil acesso ^(^ ^7^ ^)^ . 

 Em contextos de maior complexidade, como nas emergências em saúde pública e desastres, sejam de origem natural, tecnológica ou social, o transporte aéreo para remoção de pacientes busca salvar o maior número possível de vidas, além de oferecer auxílio a hospitais com demanda de grande quantidade de vítimas para atendimento ^(^ ^8^ ^)^ . 

 Diante disso, destaca-se a necessidade de formação/preparação de enfermeiros para esse tipo de atividade, posto que esses profissionais geralmente integram parte significativa das equipes de saúde de transporte aeroespacial ^(^ ^9^ ^-^ ^10^ ^)^ . Além da assistência direta propriamente dita, ressalta-se o papel de gerenciamento exercido pelo enfermeiro em desastres, voltado para a redução de potenciais riscos e agravos à saúde que possam afetar as vítimas e a própria equipe ^(^ ^11^ ^)^ . 

 Estudos sobre as competências de enfermeiros em desastres têm sido realizados em escala global ^(^ ^2^ ^,^ ^8^ ^-^ ^12^ ^)^ . Esse movimento vem mostrando a necessidade de discussão amplificada do tema e de incorporação de evidências sobre o desenvolvimento de competências específicas a serem aplicadas nas distintas tipologias de desastres. Isso implica pensar na revisão contínua e no aprimoramento de estratégias de capacitação, a fim de garantir maior operacionalidade das missões e padrão de resposta mais assertivo, sistematizado e orientado por ações de cuidado e de gerenciamento sustentadas pela ciência da Enfermagem ^(^ ^9^ ^,^ ^13^ ^)^ . 

 Assim sendo, o estudo se justifica pelas necessidades de exploração deste campo emergente e em constante evolução, de serem garantidas maior segurança e eficácia da assistência, de aprimoramento de programas de treinamento, de desenvolvimento de práticas e políticas, e inclusive de futuras pesquisas. Como ponto de partida, realizou-se uma busca preliminar em dezembro de 2023 na MEDLINE (via PubMed), *JBI Evidence Synthesis* , PROSPERO e *Cochrane Database of Systematic Reviews* , oportunidade em que não foram localizadas outras revisões sistemáticas ou de escopo em andamento ou publicadas sobre as competências do enfermeiro aeroespacial em situações de desastres. 

Diante do exposto, foi traçado o seguinte objetivo: mapear as competências do enfermeiro aeroespacial em situações de desastres.

## Método

### Tipo de estudo

 Trata-se de revisão de escopo realizada em conformidade com as recomendações da JBI ^(^ ^14^ ^)^ , cujo protocolo de pesquisa foi registrado no *Open Science Framework* : osf.io/rh2t6 e DOI: 10.17605/OSF.IO/BRY5Q. 

 A pergunta de revisão foi: quais as competências do enfermeiro aeroespacial no contexto de desastres? Definiu-se o mnemônico PCC (P – População, C – Conceito e C – Contexto) como: População - profissionais enfermeiros que atuam no cenário aeroespacial; Conceito - competências, abrangendo a mobilização de conhecimentos, habilidades e atitudes necessárias para o desempenho de atividades ou funções específicas ^(^ ^12^ ^)^ ; e Contexto - transporte aéreo em desastres, sejam eles de qualquer tipologia (natural, tecnológica ou social). 

 Por meio desses elementos, foram utilizados os vocabulários controlados: Descritores em Ciências da Saúde (DeCS), *Medical Subject Headings* (MESH) e Emtree ( *Embase subject headings* ). Após a realização da busca preliminar, foram acrescentados termos adicionais identificados nos títulos, resumos e descritores/MESH dos artigos ( Figura 1 ). 

### Critérios de elegibilidade

Considerou-se como critérios de elegibilidade as fontes de informação técnico-cientificas que abordaram as competências do enfermeiro aeroespacial em situações de desastres, em conformidade com o mnemônico PCC e a pergunta de revisão.

Foram incluídos estudos disponíveis na íntegra, sem a delimitação de balizas temporais ou linguísticas, nem pela definição de acesso aberto das fontes, com o intuito de amplificar a abrangência da pesquisa.

Publicações de quaisquer naturezas, derivadas de estudos de abordagem qualitativa, quantitativa e mista foram consideradas. Desse modo, foram analisados estudos primários, desenhos experimentais e quase experimentais, revisões, antes e depois, observacionais, séries temporais, estudos de coorte e transversais, além de casos-controle.

 Foram também previstos estudos de literatura cinzenta (banco de teses e dissertações, diretrizes, protocolos, sites, opinião e *guidelines* ). Foram excluídos livros, capítulos e cartas ao editor, bem como publicações duplicadas. 

### Período

As buscas foram realizadas de outubro a dezembro de 2023.

### Fontes de informação

 As buscas se deram no Portal Regional da Biblioteca Virtual em Saúde (BVS), de responsabilidade do Centro Latino-Americano e do Caribe de Informação em Ciências da Saúde (BIREME), onde constam bases de dados como *:* Í *ndice Bibliográfico Espa* ñ *ol en Ciencias de la Salud* (IBECS), Bases de Dados de Enfermagem (BDENF), Literatura Latino-Americana e do Caribe em Ciências da Saúde (LILACS), *Red Peruana de Bibliotecas en Salud* (LIPECS), *Scientific Electronic Library Online* (SciELO), *Medical Literature Analysis and Retrieval System* O *nline* via PubMed, da *National Library of Medicine* (NLM), entre outras. 

 Por meio do Portal de Periódicos da CAPES, as seguintes bases de dados foram acessadas: Ebsco: *Cummulative Index to Nursing and Allied Health Literature* (CINAHL) e *Academic Search Premier* (ASP); Elsevier: Embase e Scopus; *Clarivate Analytics* : *Web of Science* ; e, ainda, a *CAB Direct* (plataforma que permite pesquisar na *CAB Abstracts* ) e a *Global Health* . 

 A pesquisa incluiu também *sites* sobre enfermagem aeroespacial, socorro aeromédico, legislação profissional, *site* do *International Council of Nurses* (ICN), mecanismos de busca na internet e bibliotecas digitais que incluíssem teses e dissertações. 

 Para sistematizar a busca através da literatura cinzenta, foram considerados o portal integrador e de literatura cinzenta *Science.gov* : USA.gov, *Epistemonikos: Database of the Best Evidence-Based Health Care* , *information Technologies and a network of experts* , e *National Institute for Health and Care Excellence* (NICE). 

### Estratégias de busca

As buscas ocorreram em três etapas por dois revisores, de maneira independente. Manteve-se o processo de mascaramento entre ambos. A pesquisa contou com o apoio de um terceiro revisor para a resolução de dissidências, além de uma bibliotecária vinculada a uma universidade pública federal para orientação e acompanhamento do processo.

 A análise dos títulos, dos resumos e dos descritores foi realizada na primeira etapa, por meio de busca inicial no *Medical Literature Analysis and Retrieval System Online* (MEDLINE). Foram utilizados os operadores booleanos “AND” e “OR”, em conjunto com as palavras-chaves e descritores ( Figura 1 ). 

**Figura 1:** - Estratégias de busca aplicadas na MEDLINE

**Pesquisa**	**Estratégias de Busca**	**Resultados**
#01	**Search:** ”Nursing”[mh] OR Nursing ^*^ [tiab] OR “Nurses”[mh] ORNurse ^*^ [tiab] Sort by: Most Recent	690,130
#02	**Search:** ”Air Ambulances”[mh] OR Air Ambulance ^*^ [tiab] OR Emergency Helicopter ^*^ [tiab] OR Helicopter Ambulance ^*^ [tiab] OR”Aircraft”[mh] OR Aircraft ^*^ [tiab] OR Helicopter ^*^ [tiab] OR Airplane ^*^ [tiab] OR “Transportation of Patients”[mh] OR Patients Transportation ^*^ [tiab] OR Aeromedical evacuation[tiab] OR Airport ^*^ [tiab] OR “Aerospace Medicine”[mh] OR “Aviation Medicine”[tiab] OR “Space Medicine”[tiab] OR aerospace[tiab] OR Air[tiab] OR helicopter ^*^ [tiab] OR aircraft ^*^ [tiab] OR airplane ^*^ [tiab] Sort by: Most Recent	362,543
#03	**Search:** ”Disasters”[mh] OR Disaster ^*^ [tiab] OR Emergencies[mh] OR Emergenc ^*^ [tiab] OR Biological Disaster ^*^ [tiab] OR “Mass Casualty Incidents”[mh] OR Mass Casualty Incident ^*^ [tiab] OR Mass Casualt ^*^ [tiab] OR terror[tiab] OR Bioterrorism[tiab] OR Terrorism[tiab] Sort by: Most Recent	594,755
#04	**Search:** #01 AND #02 AND #03 Sort by: Most Recent	888

^*^ OR = ou; ^†^ AND = e

A segunda etapa foi desenvolvida a partir da busca completa em todos os bancos de dados e repositórios previamente selecionados.

Já a terceira etapa se deu a partir da análise das listas de referências de todas as fontes que se enquadraram nos critérios de elegibilidade. Ademais, buscou-se informações adicionais por meio do contato com os autores dos estudos primários, inclusive para obter acesso a outros estudos sobre o tema.

### Seleção das fontes

 Depois da realização das buscas, todas as citações identificadas foram importadas para o aplicativo *Rayyan* ^®^ ( *Qatar Computing Research Institute* , Doha, Qatar). Inicialmente, os dados foram analisados por meio de (re) leituras dos títulos, descritores e resumos, seguindo os critérios de elegibilidade. Os estudos excluídos foram apresentados no fluxograma *Preferred Reporting Items for Systematic reviews and Meta-Analyses extension for Scoping Reviews* (PRISMA-ScR) ^(^ ^15^ ^)^ quanto à população, conceito e contexto estabelecidos. 

 O gerenciador *EndNote Web* ( *Clarivate Analytics* , PA, EUA) foi utilizado para a organização das referências e identificação de estudos duplicados. 

Ambos os pesquisadores leram na íntegra os textos, os quais foram arquivados em pastas digitais. Os resultados obtidos pela seleção foram apresentados em formato de fluxograma, a partir do modelo PRISMA-ScR.

### Extração de dados

Após a leitura integral dos textos, os dados selecionados foram extraídos a partir de instrumento adaptado, conforme as recomendações do JBI.

Para a operacionalização do instrumento, um teste piloto foi realizado em três fontes, com o intuito de buscar a familiaridade dos revisores com o processo de seleção, extração e busca dos dados, oportunidade em que foram identificadas dúvidas e realizados alguns ajustes.

### Análise e apresentação de dados

 A partir dos dados extraídos, foi realizada a análise de conteúdo indutiva ^(^ ^14^ ^)^ . As informações reportaram o título do artigo/documento, autores (nome, titulação, profissão, instituição, país), descritores, tipo de fonte de informação, idioma, objetivo(s), desenho e abordagem do estudo, origem e tipologia da situação de desastre, e competências da enfermagem aeroespacial no contexto de desastres (nas fases de pré-voo, voo e pós-voo). 

A síntese dos resultados foi apresentada sob a forma de fluxo, quadros e diagrama imagético, buscando-se os devidos alinhamentos com o objetivo e a pergunta de pesquisa.

## Resultados

 Identificou-se 4.981 publicações, com 1.882 duplicadas. Assim, 3.099 publicações foram importadas para o *software Rayyan* . Após a seleção dupla cega por revisores, foram excluídas 2.765 fontes. Permaneceram 334 para análise de texto completo. Após a análise pareada e mascarada foram excluídas 30 publicações em função da população, 152 pelo conceito, e 18 pelo contexto. Restaram 37 artigos incluídos ( Figura 2 ). 

 Por outras estratégias de busca foram recuperadas 796 publicações. Destas, 130 no *Google Scholar* e 666 por busca reversa. Desse total, 12 estavam duplicadas; e 784 foram excluídas, sendo 236 pela população, 404 pelo contexto e 117 pelo conceito. Assim, não foram incluídas novas publicações por outras formas de busca, pois não foram evidenciadas fontes que abordassem a temática das competências do enfermeiro aeroespacial no contexto de desastres, em conformidade com os critérios de elegibilidade. Ao todo, foram então incluídas 37 publicações para revisão ( Figura 2 ). 

 Dos 37 estudos sobre as competências do enfermeiro aeroespacial no contexto de desastres, que passaram a ser publicados a partir de 1991, identificou-se uma média de dois a três artigos publicados por ano. Destaca-se que em 2010, 2011, 2017 e 2020 identificou-se três publicações em cada ano; já em 2003, 2005, 2007, 2008, 2012, 2013, 2021, 2022 e 2023 ocorreram duas publicações por ano, conforme apresentado na Figura 3 . 

Guardadas as devidas proporções, esse resultado pode estar relacionado aos avanços que passaram a ocorrer no campo da Enfermagem no que diz respeito ao emprego de práticas assistenciais no âmbito dos sistemas de aviação, especialmente no que se refere ao aumento da frequência e da complexidade dos desastres, da percepção mais clara de risco frente a tais eventos, e da necessidade de discussão de abordagens de múltiplas vítimas por casos clínico-cirúrgicos complexos, que exigem o transporte e resgate aeromédico em tais situações.

 Destaca-se que o advento da especialidade da enfermagem aeroespacial contribui para a necessidade de disseminação científica da temática e, por conseguinte, de sistematização desta prática. Registra-se, ainda, o movimento do ICN iniciado mais enfaticamente no ano de 2009, em buscar apontar estratégias de formalização de competências de enfermeiros para atuação em desastres ^(^ ^10^ ^,^ ^16^ ^-^ ^17^ ^)^ . 

**Figura 2:** - Fluxograma PRISMA-ScR

 Quanto ao país, identificou-se que 23 estudos foram originados dos Estados Unidos da América (EUA), 11 do Brasil, um da China, um da Finlândia e um da Irlanda. Sobre o idioma, 26 foram redigidos em inglês e 11 em português. Ressalta-se o investimento no transporte e resgate aeromédico em âmbitos civil e militar nesses países ( Figura 3 ). 

 Identificou-se que sete artigos foram publicados no periódico *Air Medical Journal* , seis na Revista Brasileira de Enfermagem, três no *Critical Care Nursing Clinics* , dois no *Military Medicine* e dois na revista Texto & Contexto – Enfermagem ( Figura 3 ). 

**Figura 3:** - Caracterização das publicações mapeadas

**Nº**	**Ano**	**Tipo de desastre**	**Fonte de informação**	**País**	**Idioma**
F1 ^(^ ^18^ ^)^	1991	Não tipificado	Rev Bras Enferm	Brasil	Português
F2 ^(^ ^19^ ^)^	1995	Não tipificado	*Air Medical Journal*	Estados Unidos	Inglês
F3 ^(^ ^20^ ^)^	1996	Não tipificado	*Air Medical Journal*	Estados Unidos	Inglês
F4 ^(^ ^21^ ^)^	1997	Guerra	Rev da Escola de Enfermagem da USP	Brasil	Português
F5 ^(^ ^22^ ^)^	1999	Guerra	Acta Paul Enferm	Brasil	Português
F6 ^(^ ^23^ ^)^	2003	Não tipificado	Rev Bras Enferm	Brasil	Português
F7 ^(^ ^24^ ^)^	2003	Não tipificado	*Critical Care Nursing Clinics*	Estados Unidos	Inglês
F8 ^(^ ^25^ ^)^	2005	Não tipificado	*Critical Care Medicine*	Estados Unidos	Inglês
F9 ^(^ ^26^ ^)^	2005	Tsunami	*Scandinavian Journal of Surgery*	Finlândia	Inglês
F10 ^(^ ^27^ ^)^	2007	Não tipificado	*AORN Journal*	Estados Unidos	Inglês
F11 ^(^ ^28^ ^)^	2007	Furacão Katrina	*Disaster Management & Response*	Estados Unidos	Inglês
F12 ^(^ ^29^ ^)^	2008	Tsunami	*Air Medical Journal*	Estados Unidos	Inglês
F13 ^(^ ^30^ ^)^	2008	Guerra	*Critical Care Nursing Clinics of North America*	Estados Unidos	Inglês
F14 ^(^ ^31^ ^)^	2010	Não tipificado	*Critical Care Nurse*	Estados Unidos	Inglês
F15 ^(^ ^32^ ^)^	2010	Não tipificado	*Journal of Trauma Nursing| JTN*	Estados Unidos	Inglês
F16 ^(^ ^33^ ^)^	2010	Guerra	*International Journal of Nursing Practice*	Estados Unidos	Inglês
F17 ^(^ ^34^ ^)^	2011	Não tipificado	Rev Bras Enferm	Brasil	Português
F18 ^(^ ^35^ ^)^	2011	Não tipificado	Texto & Contexto –Enfermagem	Brasil	Português
F19 ^(^ ^36^ ^)^	2011	Não tipificado	Rev Bras Enferm	Brasil	Português
F20 ^(^ ^37^ ^)^	2012	Não tipificado	Rev Bras Enferm	Brasil	Português
F21 ^(^ ^38^ ^)^	2012	Guerra	*Air Medical Journal*	Estados Unidos	Inglês
F22 ^(^ ^39^ ^)^	2013	Não tipificado	*Journal of Emergency Nursing*	Estados Unidos	Inglês
F23 ^(^ ^40^ ^)^	2013	Guerra	*Journal of Emergency Nursing*	Estados Unidos	Inglês
F24 ^(^ ^41^ ^)^	2015	Guerra	*Journal of Emergency Nursing*	Estados Unidos	Inglês
F25 ^(^ ^42^ ^)^	2017	Não tipificado	*International Emergency Nursing*	Estados Unidos	Inglês
F26 ^(^ ^4^ ^)^	2017	Guerra	Esc Anna Nery – Revista de Enfermagem	Brasil	Português
F27 ^(^ ^43^ ^)^	2017	Não tipificado	*Military Medicine*	Estados Unidos	Inglês
F28 ^(^ ^44^ ^)^	2018	QBRN ^*^	*Air Medical Journal*	Estados Unidos	Inglês
F29 ^(^ ^45^ ^)^	2019	Guerra e desastres de origem natural	*Nursing Outlook*	Estados Unidos	Inglês
F30 ^(^ ^46^ ^)^	2020	Não tipificado	*Military Medicine*	Estados Unidos	Inglês
F31 ^(^ ^47^ ^)^	2020	Não tipificado	*Nurse Leader*	Estados Unidos	Inglês
F32 ^(^ ^48^ ^)^	2020	Não tipificado	*Scandinavian Journal of Trauma, Resuscitation and Emergency Medicine country*	Irlanda	Inglês
F33 ^(^ ^49^ ^)^	2021	Não tipificado	*Air Medical Journal*	Estados Unidos	Inglês
F34 ^(^ ^9^ ^)^	2021	Não tipificado	*Nursing*	Brasil	Português
F35 ^(^ ^50^ ^)^	2022	Não tipificado	*Hong Kong Journal of Emergency Medicine*	China	Inglês
F36 ^(^ ^51^ ^)^	2023	Não tipificado	*Air Medical Journal*	Estados Unidos	Inglês
F37 ^(^ ^7^ ^)^	2023	QBRN ^*^	Texto & Contexto *–* Enfermagem	Brasil	Português

^*^ QBRN = Químicos, biológicos, radiológicos e nucleares

 No que se refere à tipologia dos desastres, identificou-se que 22 artigos abordaram o contexto de desastres de modo geral, sem tipificá-lo; nove referiam-se às guerras, dois a eventos envolvendo agentes químicos, biológicos, radiológicos e nucleares (QBRN), dois a eventos geológicos (tsunami), um meteorológico (furacão) e um relacionou-se à origem natural (sem definir a subtipologia exata) ( Figura 3 ). 

Essa pluralidade de tipos de eventos sugere a necessidade de maior exploração do tema em questão, na compreensão de que as especificidades de cada evento podem implicar em práticas orientadas para as situações clínicas particularizadas. Ademais, compreender os desafios enfrentados e as lições aprendidas em tais eventos é estratégico na assunção de ações de gestão do risco de desastres, o que implica pensar em processos, ou seja, em ações a serem desenvolvidas antes, durante e após tais eventos. Observa-se, ainda, certa ênfase em situações de guerras e eventos QBRN entre os estudos mapeados, o que evoca reflexões sobre as preocupações contemporâneas desse tipo de evento.

 Em síntese, as competências do enfermeiro aeroespacial no contexto de desastres, que foram mapeadas por esta revisão de escopo, foram elencadas em três categorias, a saber: pré-voo, voo e pós-voo ( Figura 4 ). 

**Figura 4:** - Competências do enfermeiro aeroespacial no contexto de desastres

**Fase do transporte**	**Mapeamento das competências do enfermeiro aeroespacial no contexto de desastres**
**PRÉ-VOO**	Conhecer a fisiologia do voo e os procedimentos de emergência aeronáutica para prestar assistência de enfermagem de qualidade ^(^ ^7^ ^,^ ^9^ ^,^ ^19^ ^,^ ^33^ ^,^ ^36^ ^-^ ^37^ ^)^ .
	Conhecer práticas de segurança de voo, do paciente e do profissional relacionadas à aeronave em solo ou em voo ^(^ ^9^ ^,^ ^33^ ^,^ ^36^ ^-^ ^37^ ^,^ ^45^ ^-^ ^48^ ^)^ .
	Conhecer as aeronaves utilizadas em transporte aéreo, e compreender o uso de equipamentos médicos relacionados ^(^ ^9^ ^,^ ^19^ ^,^ ^24^ ^,^ ^27^ ^,^ ^29^ ^,^ ^33^ ^,^ ^50^ ^)^ .
	Saber realizar o preparo da aeronave para a missão ^(^ ^9^ ^,^ ^19^ ^,^ ^24^ ^,^ ^27^ ^,^ ^46^ ^,^ ^49^ ^-^ ^50^ ^)^ .
	Realizar o *checklist* dos materiais e medicamentos padronizados para uso na aeronave ^(^ ^9^ ^,^ ^19^ ^,^ ^24^ ^,^ ^27^ ^,^ ^49^ ^-^ ^50^ ^)^ .
	Prover os materiais, equipamentos e oxigênio necessários ^(^ ^9^ ^,^ ^19^ ^,^ ^24^ ^,^ ^27^ ^,^ ^49^ ^-^ ^50^ ^)^ .
	Possuir perfil de liderança e capacidade de tomada de decisão ^(^ ^28^ ^,^ ^45^ ^)^ .
	Atuar como profissional formador, desenvolvendo programas em equipe para assegurar que a equipe possua habilidades, competências e conhecimentos atualizados ^(^ ^45^ ^)^ .
	Possuir habilidades técnicas para lidar com desastres, habilidades de comunicação e capacidade de lidar com múltiplas vítimas ^(^ ^9^ ^,^ ^19^ ^,^ ^24^ ^,^ ^27^ ^,^ ^34^ ^)^ .
	Possuir boa comunicação oral e escrita, para discutir casos com a equipe e realizar os relatórios necessários ^(^ ^7^ ^,^ ^35^ ^,^ ^37^ ^,^ ^45^ ^)^ .
	Realizar a verificação da necessidade do uso do aeronave, identificando o caráter da emergência por meio do estado, quantidade de vítimas e local da ocorrência ^(^ ^9^ ^,^ ^18^ ^,^ ^36^ ^,^ ^44^ ^)^ .
	Planejar, junto com a equipe multiprofissional, toda a missão ^(^ ^22^ ^,^ ^36^ ^,^ ^44^ ^,^ ^51^ ^)^ .
	Realizar a avaliação do paciente (Histórico de Enfermagem) e participar da decisão sobre a indicação de remoção ou não do paciente por meio aéreo ^(^ ^20^ ^,^ ^25^ ^,^ ^35^ ^,^ ^38^ ^)^ .
	Realizar procedimentos de diversas complexidades no preparo do paciente para o transporte, de acordo com o quadro clínico ^(^ ^7^ ^,^ ^9^ ^,^ ^22^ ^-^ ^23^ ^,^ ^25^ ^,^ ^27^ ^-^ ^28^ ^,^ ^30^ ^,^ ^49^ ^-^ ^50^ ^)^ .
**VOO**	Possuir conhecimento sobre os procedimentos de emergência da aeronave em que realiza o transporte ^(^ ^25^ ^-^ ^26^ ^)^ .
	Ter a capacidade de desenvolver e gerenciar ações do plano de resposta de desastres em ambiente aéreo ^(^ ^9^ ^,^ ^27^ ^,^ ^39^ ^)^ .
	Possuir boa comunicação escrita para realizar registros e anotações de Enfermagem ^(^ ^21^ ^-^ ^22^ ^)^ .
	Possuir capacidade de liderança da equipe de cuidados durante o voo ^(^ ^22^ ^)^ .
	Gerenciar o fluxo de pacientes, recursos humanos, materiais, equipamentos e suprimentos durante o voo ^(^ ^9^ ^,^ ^19^ ^,^ ^22^ ^,^ ^26^ ^-^ ^28^ ^,^ ^31^ ^-^ ^32^ ^,^ ^42^ ^,^ ^44^ ^)^ .
	Possuir capacidade de gerenciar as intercorrências durante o voo ^(^ ^22^ ^,^ ^36^ ^)^ .
	Utilizar os instrumentos que garantam a segurança e a saúde profissional (EPI e EPC) ^(^ ^7^ ^,^ ^40^ ^)^ .
	Realizar anamnese e exame físico direcionado, estabelecendo o processo de Enfermagem ^(^ ^20^ ^,^ ^25^ ^,^ ^36^ ^,^ ^38^ ^-^ ^39^ ^,^ ^41^ ^)^ .
	Oferecer apoio psicoemocional ao paciente durante o transporte ^(^ ^4^ ^,^ ^21^ ^-^ ^22^ ^,^ ^36^ ^,^ ^42^ ^)^ .
	Realizar procedimentos de diversas complexidades durante o voo, de acordo com o quadro clínico do paciente ^(^ ^7^ ^,^ ^9^ ^,^ ^22^ ^-^ ^23^ ^,^ ^25^ ^,^ ^27^ ^-^ ^30^ ^,^ ^36^ ^,^ ^39^ ^,^ ^41^ ^,^ ^48^ ^-^ ^50^ ^)^ .
	Realizar intubação orotraqueal, em casos necessários ^(^ ^51^ ^)^ .
**PÓS-VOO**	Possuir boa comunicação escrita para realizar o preenchimento da documentação do paciente em formulário ou sistema próprio, organizar e protocolar os registros em ordem cronológica dos acontecimentos ^(^ ^19^ ^,^ ^22^ ^,^ ^26^ ^,^ ^35^ ^,^ ^40^ ^)^ .
	Desenvolver comunicação técnica clara por meio da descrição detalhada do caso do paciente para a equipe de saúde em solo, indicando todas as alterações e intervenções realizadas no pré-voo e durante o voo ^(^ ^19^ ^,^ ^22^ ^,^ ^26^ ^,^ ^40^ ^)^ .
	Realizar a desinfecção dos equipamentos de saúde, e repor a previsão de materiais para a próxima missão ^(^ ^22^ ^)^ .

## Discussão

 Esta revisão de escopo possibilitou mapear as competências do enfermeiro aeroespacial no contexto de desastres, as quais foram apresentadas de forma sistematizada em três fases: pré-voo (12 competências), voo (11 competências) e pós-voo (três competências), no sentido de instruir profissionais, gestores, educadores e pesquisadores ^(^ ^4^ ^,^ ^7^ ^,^ ^9^ ^,^ ^18^ ^-^ ^51^ ^)^ . 

 Evidenciou-se que o desenvolvimento dessas competências demanda uma pluralidade de conhecimentos, habilidades e atitudes específicas para a assistência e gestão do cuidado em cenários com múltiplos e diferentes desafios e níveis de complexidade, ameaças, vulnerabilidades e riscos ^(^ ^4^ ^,^ ^7^ ^,^ ^9^ ^,^ ^18^ ^-^ ^51^ ^)^ . 

 Em desastres, pode ser necessário o emprego de aeronaves para o resgate e transporte de pacientes de diferentes modelos e tamanhos, de asas rotativas e fixas. Nesse sentido, a revisão mostrou que o enfermeiro aeroespacial possui papel estratégico junto à equipe de voo na tomada de decisão sobre qual tipo de aeronave é o mais adequado para cada situação ^(^ ^18^ ^-^ ^19^ ^,^ ^36^ ^,^ ^44^ ^)^ . 

 Cabe ao enfermeiro aeroespacial avaliar, junto à equipe de voo, as condições do local do resgate, a gravidade do evento, o número de vítimas a serem transportadas, as necessidades individuais de cada paciente e os elementos envolvidos para possibilitar o transporte e resgate das vítimas de forma a manter a segurança e integridade das vítimas e da equipe na aeronave ^(^ ^18^ ^-^ ^19^ ^,^ ^36^ ^,^ ^44^ ^)^ . 

 Em geral, as aeronaves de asas rotativas percorrem curtas distâncias, têm maior condição de acesso ao local de origem dos pacientes e permitem frequentemente o transporte em baixas altitudes, diferentemente das de asas fixas, que percorrem longas distâncias, transportam maior número de vítimas não infecciosas, além de alcançarem maiores altitudes ^(^ ^9^ ^,^ ^18^ ^,^ ^44^ ^)^ . 

 Evidenciou-se que o enfermeiro aeroespacial deve dispor de conhecimentos específicos sobre fisiologia de voo, com o objetivo de prever e identificar, de forma imediata, eventuais riscos ou mudanças no quadro clínico do paciente oriundas do ambiente hipobárico. Como exemplo, cita-se a hipóxia de altitude, os efeitos da pressurização da cabine, a farmacocinética e farmacodinâmica clínica em ambiente de voo e as alterações no padrão dos equipamentos ^(^ ^7^ ^,^ ^9^ ^,^ ^19^ ^,^ ^34^ ^)^ . 

 Ademais, são necessários conhecimentos sobre procedimentos de segurança de voo e de emergências aeronáuticas, com a premissa de mitigar situações oriundas da velocidade da aeronave, ruídos, vibrações e das forças gravitacionais, desenvolver conhecimento sobre o correto posicionamento dos pacientes, e perceber risco de deslocamento de materiais e equipamentos que pode ocorrer durante a decolagem e o pouso e, eventualmente, provocar acidentes ^(^ ^7^ ^,^ ^9^ ^,^ ^19^ ^,^ ^34^ ^)^ . 

 No transporte e resgate aéreo é essencial que o enfermeiro possua habilidades técnicas que abranjam o conhecimento prévio e bem sustentado sobre os fenômenos de desastres e suas diferentes tipologias (guerras, atentados terroristas, inundações, deslizamentos de terra, ondas de calor e de frio, epidemias, pragas, acidentes com materiais QBRN, terremotos, tsunamis, entre outros), além da habilidade em lidar com múltiplas vítimas, uma situação recorrente em tais eventos ^(^ ^9^ ^,^ ^19^ ^,^ ^24^ ^,^ ^27^ ^,^ ^34^ ^)^ . Com efeito, ao compreender o contexto, a magnitude e as particularidades dos desastres, o enfermeiro aeroespacial terá melhores condições de preparar a aeronave e os equipamentos para a missão. 

 Para a realização desta atividade com êxito, é necessário o uso de *checklists* de materiais, insumos, medicamentos e demais suprimentos necessários, que são padronizados para a aeronave, o tipo de desastre, bem como para a complexidade e o tempo previsto da missão. Esses itens são essenciais para que o cuidado aos pacientes durante o voo seja realizado sem intercorrências, no sentido de evitar a falta de algum material ou equipamento e manter adequadas as condições de uso e funcionamento dos materiais e equipamentos. Assim, assegura-se que os procedimentos serão realizados de forma correta, maximizando o potencial de benefício aos pacientes ^(^ ^7^ ^,^ ^9^ ^,^ ^22^ ^-^ ^23^ ^,^ ^25^ ^,^ ^27^ ^,^ ^29^ ^-^ ^30^ ^,^ ^49^ ^-^ ^50^ ^)^ . 

 Os estudos destacaram a necessidade do planejamento do transporte, resgate aéreo ^(^ ^18^ ^,^ ^41^ ^,^ ^47^ ^)^ e cuidado a partir do estabelecimento do Processo de Enfermagem, com a sustentação de teorias de Enfermagem ^(^ ^52^ ^-^ ^53^ ^)^ . Também apontaram a necessidade de criatividade e utilização de instrumentos e tecnologias, tais como aplicativos e inteligência artificial, para promover, em curto tempo e em cenários de alta complexidade ^(^ ^53^ ^)^ , a anamnese e o exame físico do paciente, o estabelecimento de diagnósticos de Enfermagem prioritários e as intervenções essenciais a serem executadas e adequadamente mantidas antes, durante e após o voo, com o propósito de superar os desafios oriundos do ambiente aéreo ^(^ ^7^ ^,^ ^18^ ^-^ ^19^ ^,^ ^23^ ^,^ ^26^ ^,^ ^33^ ^,^ ^43^ ^)^ . 

 As habilidades técnicas, juntamente com as capacidades de comunicação para lidar com múltiplas vítimas, são realçadas por diversos estudos ^(^ ^7^ ^,^ ^9^ ^,^ ^22^ ^-^ ^23^ ^,^ ^25^ ^,^ ^27^ ^-^ ^28^ ^,^ ^30^ ^,^ ^46^ ^,^ ^49^ ^)^ . Também foi amplamente destacada, a partir da variedade de procedimentos a serem realizados pelo enfermeiro, a monitorização constante dos pacientes, o que abrange a oximetria de pulso e a administração de oxigênio para prevenir a hipóxia de altitude em casos necessários ^(^ ^7^ ^,^ ^9^ ^,^ ^22^ ^-^ ^23^ ^,^ ^25^ ^,^ ^27^ ^-^ ^28^ ^,^ ^30^ ^,^ ^40^ ^,^ ^46^ ^)^ . Outros procedimentos essenciais incluem a atenção ao padrão ventilatório, a observação de sinais de pneumotórax e a manutenção de drenos de tórax em pleno funcionamento. Adicionalmente, a administração de medicamentos, a preparação para situações de agitação psicomotora e a prevenção de crises convulsivas ^(^ ^9^ ^,^ ^19^ ^,^ ^22^ ^,^ ^26^ ^-^ ^28^ ^,^ ^30^ ^,^ ^32^ ^,^ ^42^ ^-^ ^43^ ^)^ . 

 Destaca-se que o enfermeiro aeroespacial frequentemente realizará procedimentos técnicos durante a fase de pré-voo, para evitar executá-los em um ambiente ruidoso, com vibrações e reduzida iluminação produzidos pelo voo. Porém, em casos necessários, os procedimentos deverão ser realizados durante o voo, com a adoção de cuidados redobrados que preservem a segurança do paciente, do profissional e da aeronave ^(^ ^9^ ^,^ ^19^ ^,^ ^22^ ^,^ ^26^ ^-^ ^28^ ^,^ ^30^ ^,^ ^32^ ^,^ ^42^ ^-^ ^43^ ^)^ . 

 As fontes mostraram a atuação do enfermeiro em intubações orotraqueais no contexto aeroespacial, em casos necessários ^(^ ^51^ ^)^ . Com efeito, tal prática foi registrada nos EUA, onde a legislação respalda o enfermeiro na realização desse tipo de procedimento ^(^ ^54^ ^)^ . 

 As fontes enfatizam também habilidades relacionadas à liderança, comunicação efetiva, práticas de segurança, organização de equipes e gestão de recursos durante o voo ^(^ ^9^ ^,^ ^19^ ^,^ ^24^ ^,^ ^27^ ^,^ ^34^ ^)^ . Em desastres, o enfermeiro aeroespacial precisa desenvolver habilidades voltadas ao gerenciamento de equipes para garantir resposta eficaz e rápida ^(^ ^9^ ^,^ ^19^ ^,^ ^24^ ^,^ ^27^ ^,^ ^34^ ^)^ . A capacidade de tomar decisões bem sustentadas e assertivas é fundamental para lidar com situações imprevisíveis. Ademais, em ambientes com recursos limitados, saber gerenciar materiais, equipamentos e pessoal é vital para garantir que todos os recursos disponíveis sejam utilizados de forma otimizada e mantenham-se operantes ^(^ ^28^ ^,^ ^45^ ^)^ . 

 Em geral, os fenômenos de desastres podem gerar danos de diferentes naturezas aos pacientes, relacionados a múltiplas lesões, contusões, fraturas, lacerações, amputações, hemorragias, ferimentos por armamento, intoxicações, lesões respiratórias, infecções, hiper/hipotermia, queimaduras, desidratação, exaustão, inclusive estresse agudo e transtorno de estresse pós-traumático ^(^ ^35^ ^)^ . Cabe ao enfermeiro aeroespacial estar preparado para lidar com as particularidades dessas ocorrências, garantindo que o paciente receba as intervenções de Enfermagem necessárias até chegar ao hospital de destino ^(^ ^35^ ^)^ . 

 Ademais, a segurança do paciente é uma questão consideravelmente abordada no contexto da enfermagem aeroespacial. Desse modo, o enfermeiro precisa (re) conhecer as práticas de segurança relacionadas às aeronaves em solo ou em voo, para poder intervir, promovendo e provendo segurança ^(^ ^9^ ^,^ ^37^ ^,^ ^45^ ^,^ ^47^ ^-^ ^48^ ^)^ . 

 No campo acadêmico, destaca-se que a busca pela excelência na formação profissional, nos âmbitos de graduação e pós-graduação *lato sensu* e em cursos de formação de enfermeiros militares, tem o potencial de aprimorar a segurança durante os voos, assegurar a proteção da equipe transportada, otimizar o uso do tempo e dos recursos necessários, e, sobretudo, oferecer uma assistência de alta qualidade, sustentável e adequada para a condução de transporte aéreo de pacientes ^(^ ^7^ ^,^ ^45^ ^)^ . 

 Além disso, o conhecimento sobre segurança de voo e de possíveis intercorrências em aeronaves auxilia a garantir a própria segurança do enfermeiro aeroespacial e dos demais membros da equipe de voo, como nos casos de despressurização da cabine e de turbulência severa. Isso inclui saber como agir nesses casos emergenciais, como utilizar os equipamentos de segurança previstos e seguir os protocolos de evacuação emergencial da aeronave, caso necessário, o que tende a permitir uma colaboração mais efetiva com a tripulação aérea ^(^ ^9^ ^,^ ^37^ ^,^ ^45^ ^,^ ^47^ ^-^ ^48^ ^)^ . 

 Esses achados revelam a diversidade de competências exigidas dos enfermeiros aeroespaciais em situações críticas, mostrando a necessidade de um amplo conjunto de conhecimentos técnicos, habilidades de comunicação, gestão e liderança para garantir cuidados de alta qualidade e segurança durante o planejamento do transporte aéreo de pacientes, o que comumente torna-se mais complexo em contextos de desastres ^(^ ^9^ ^,^ ^19^ ^,^ ^24^ ^,^ ^27^ ^,^ ^34^ ^)^ . 

 Durante os resgates, muitos dos feridos se encontram com desconfortos físicos e psicossociais. O manejo da dor, náusea e ansiedade/angústia competem ao enfermeiro aeroespacial, bem como a promoção do conforto do paciente, sendo esta uma ferramenta eficaz para que os efeitos dessas ocorrências clínicas sejam minorados ou controlados ^(^ ^29^ ^)^ . 

 Na fase de pós-voo, as competências enfatizaram a importância da comunicação escrita, documentação adequada e manutenção dos equipamentos ^(^ ^19^ ^,^ ^22^ ^,^ ^26^ ^,^ ^35^ ^)^ . Tais aspectos destacam a necessidade de uma abordagem meticulosa e organizada nesta fase, assegurando não apenas a documentação precisa e clara, mas também o zelo com os equipamentos e materiais a fim de garantir a continuidade e a segurança do serviço em futuras operações de transporte aéreo de pacientes ^(^ ^22^ ^)^ . 

 Ao término do resgate, os procedimentos relacionados ao registro de Enfermagem continuam. Para tanto, o enfermeiro aeroespacial deve possuir conhecimento e oratória adequada, a fim de garantir boa comunicação, de forma técnica e clara, na descrição dos casos dos pacientes e na indicação cronológica de todas as alterações e procedimentos/cuidados realizados durante o voo ^(^ ^19^ ^,^ ^22^ ^,^ ^24^ ^,^ ^35^ ^)^ . Ao final, o enfermeiro aeroespacial realiza a desinfecção dos equipamentos, solicita e repõe os materiais necessários para a próxima missão ^(^ ^22^ ^)^ . 

 Com base nos achados, nota-se que a amplitude e a complexidade dessas competências (antes, durante e pós-voo) implicam em pensar no desenvolvimento de inovações e tecnologias de cuidado, ensino e gestão, que possam ser sistematicamente empregadas nos processos de preparação dos enfermeiros, com o intuito de potencializar a operacionalização de ações assistenciais e gerenciais ^(^ ^19^ ^,^ ^22^ ^,^ ^24^ ^,^ ^35^ ^)^ . 

 Registra-se que, em função do caos das situações de desastres, há uma certa tendência de serem indevidamente registradas as ocorrências nas três fases de cuidados de enfermagem aeroespacial. Contudo, assevera-se a necessidade do exercício dessa competência por ocasião da realização do histórico de Enfermagem, na compreensão de que tais informações são cruciais para o julgamento clínico, identificação dos diagnósticos e implementação das intervenções de Enfermagem, além da contribuição para a tomada de decisão referente à indicação de remoção ou não do paciente por meio aéreo ^(^ ^20^ ^,^ ^25^ ^,^ ^38^ ^)^ , sem contar o repasse necessário de intercorrências durante o voo para as equipes terrestres ^(^ ^19^ ^,^ ^22^ ^,^ ^26^ ^,^ ^35^ ^)^ . 

 A produção científica na área de enfermagem aeroespacial mostrou que alguns países, tais como os EUA, possuem padronização de diferentes atividades do enfermeiro. No Brasil, é uma área ainda em expansão, muito influenciada pelo modelo militar, e que carece de maior divulgação, legalização e legitimação das atividades, especialmente ao serem considerados os contextos dinâmicos e complexos de desastres, que requerem a assunção de medidas orientadas por evidências científicas e conhecimentos assentados em práticas (inter) disciplinares e intersetoriais eficazes e sistematizadas ^(^ ^4^ ^,^ ^7^ ^,^ ^9^ ^,^ ^18^ ^,^ ^21^ ^-^ ^23^ ^,^ ^34^ ^-^ ^37^ ^)^ . 

 Notavelmente, a especialidade da enfermagem aeroespacial foi reconhecida no Brasil pela resolução do Conselho Federal de Enfermagem (COFEN) nº581/2018 (alterada pela resolução COFEN nº264/2023) ^(^ ^55^ ^)^ . Assim, por meio desta previsão legal, trata-se de uma especialidade que carece de avanços de cunho legal e reconhecimento no campo civil no contexto brasileiro, o que tem o efeito de mostrar a necessidade de mais investigações científicas com vias a consolidar os fundamentos da mesma. 

Registra-se que esta revisão apresentou como limitações a dificuldade de recuperar algumas publicações, o que foi contornado, em parte, a partir de outras formas de busca, como o contato direto com autores por meio eletrônico e com bibliotecas de universidades, o que oportunizou acessar 15 dos 37 artigos na íntegra. No que tange às outras formas de busca, não foram incluídos novos artigos por não abordarem competências da enfermagem aeroespacial no contexto de desastres. Neste caso, 12 publicações já haviam sido previamente incluídas no estudo.

Diante das complexidades inerentes a essas situações, o agrupamento das competências identificadas revela a importância do domínio da fisiologia do voo, dos procedimentos de emergências aeronáuticas, das habilidades de comunicação, da liderança e das responsabilidades na preparação e atuação durante e após o voo. Este mapeamento contribui não apenas para a compreensão mais clara do papel do enfermeiro aeroespacial, mas também para o desenvolvimento de estratégias eficazes de formação, capacitação e gestão de crises nesse contexto desafiador orientadas pelas competências e pelas tipologias dos desastres.

## Conclusão

O mapeamento das competências do enfermeiro aeroespacial em desastres determinou a identificação e a compreensão mais ampliada das habilidades técnicas, dos conhecimentos específicos e das atitudes necessárias para aprimorar a assistência eficaz e segura durante as fases de pré-voo, voo e pós-voo.

Diante das demandas enfrentadas pelo enfermeiro aeroespacial em situações de desastres, há que se compreender a necessidade de desenvolvimento de um conjunto abrangente de competências, para garantir assistência de alta qualidade aos pacientes durante todas as fases do transporte aéreo. O destaque recai sobre a urgência na avaliação da necessidade de utilização de aeronaves, demandando habilidades técnicas, conhecimento da fisiologia do voo e procedimentos de emergências aeronáuticas. Em suma, a atuação do enfermeiro aeroespacial em desastres, durante o transporte aéreo, exige uma combinação de competências técnicas que envolvem o cuidado, a liderança, a comunicação e a gestão, evidenciando a necessidade contínua de formação e atualização para garantir a excelência na assistência e a segurança tanto do paciente quanto da equipe envolvida nessas situações desafiadoras. Isso implica pensar no desenvolvimento de tecnologias de cuidado, ensino e gestão, bem como em marcos regulatórios e previsões legais para suporte jurídico a esses profissionais imbuídos de salvar/manter vidas. Sugere-se estudos futuros para validar tais competências entre enfermeiros aeroespaciais e pesquisadores em contexto de desastres, a fim de aprimorar a capacidade de resposta diante desses fenômenos.
